# Valorization of Construction and Demolition Wastes and Industrial By-Products in Sustainable Concrete: Comparative Mechanical Performance of Slag Slurry-Treated Recycled Aggregate Concretes

**DOI:** 10.3390/ma19122619

**Published:** 2026-06-17

**Authors:** Hasan Yildirim, Olcay Gürabi Aydoğan, Nilufer Ozyurt, Turan Ozturan

**Affiliations:** 1 Department of Civil Engineering and Management, University of Manchester, Manchester M13 9PL, UK; 2 Department of Civil Engineering, Boğaziçi University, Istanbul 34342, Türkiye; olcay.aydogan@bogazici.edu.tr (O.G.A.); nilufer.ozyurt@bogazici.edu.tr (N.O.); ozturan@bogazici.edu.tr (T.O.)

**Keywords:** recycled aggregates, sustainable concrete, slag slurry treatment, mechanical properties, code-based prediction models

## Abstract

This study investigates the valorization of construction and demolition (C&D) waste streams and an industrial by-product for sustainable concrete production. Recycled concrete aggregates (RCA) and recycled brick aggregates (RBA), derived from C&D wastes, together with pelletized recycled fly ash aggregates (FAA) produced from thermal power plant fly ash, were used as total replacements for natural coarse aggregates. Six concrete mixtures were prepared at a constant water-to-cement ratio of 0.50 using untreated and slag slurry–treated aggregates. A slag slurry-based two-stage mixing approach (TSMA), incorporating ground granulated blast furnace slag (GGBFS), was applied as a practical and potentially scalable treatment method to enhance aggregate quality and interfacial bonding. The results show that complete replacement of natural aggregates reduced fresh concrete unit weight by up to 17%, while meeting the minimum compressive strength requirements for structural applications. Slag slurry treatment led to statistically significant improvements in mechanical properties, reduced variability, and enhanced overall reliability. In addition, widely used code-based prediction models (TS500, ACI, Eurocode-2, NZS 3101-1:2006, and CSA A23.3-04), originally developed for conventional concrete, were evaluated for their applicability in estimating key mechanical properties of recycled and by-product aggregate concretes, and alternative regression-based models were developed to improve prediction accuracy. Overall, the findings demonstrate the potential for effective utilization of C&D wastes and industrial by-products in structural concrete, contributing to resource efficiency and reduced reliance on natural aggregates.

## 1. Introduction

The swift pace of urbanization and industrialization has led to the generation of substantial volumes of construction and demolition waste (CDW) globally, with a marked impact on developing nations. The elimination of such materials continuously triggers both environmental degradation and significant landfill shortages [1,2,3,4,5]. Therefore, repurposing and recycling CDW is essential not only for safeguarding the environment but also for ensuring the sustainable consumption of raw materials [6,7,8,9,10,11]. From this point of view, the drive to minimize the construction industry’s consumption of natural raw materials has fueled extensive research into recycled aggregate concrete, capitalizing on the immense potential of CDW derivatives as viable substitutes [12,13,14,15,16,17,18,19,20,21,22,23,24,25]. Despite the ecological advantages of utilizing CDW as recycled aggregate, the presence of residual cement paste attached to these particles introduces critical material flaws. Specifically, this adhered mortar significantly elevates the porosity and water absorption of the mixture, which in turn diminishes the overall mechanical strength of the concrete [26,27]. To address these drawbacks, previous studies predominantly identify three major technical strategies designed to enhance both the properties of the recycled aggregates and the overall behavior of the resulting concrete: (i) removal of residual cement mortar [28,29,30,31,32,33,34,35,36,37], (ii) coating of recycled aggregates [33,35,37,38,39,40,41,42,43,44,45], and (iii) multi-step concrete mixing methods with pozzolanic admixtures [36,37,44,46,47,48,49,50,51,52,53,54,55,56,57,58]. Techniques such as stripping away the residual mortar or applying surface coatings are frequently hindered by high costs, extended processing times, and additional ecological footprints. Consequently, recent studies highlight that utilizing multi-stage mixing protocols combined with pozzolanic additives serves as a far more practical and efficient alternative [42,50,59,60,61,62].

Developing eco-friendly and sustainable infrastructure has remained a formidable challenge for the construction sector over the years. To overcome this hurdle, investigators have also sought practical ways to incorporate industrial by-products, namely fly ash (FA) and ground granulated blast furnace slag (GGBFS), into building materials. It is well documented that the building industry serves as a massive sink for FA and GGBFS, which are generated by coal-fired power plants and iron smelting facilities, respectively. Their diverse structural applications encompass everything from pavement sub-bases and subgrade stabilization to the execution of embankments and backfills, alongside their crucial roles in manufacturing structural blocks, ceramics, artificial aggregates, alkali-activated geopolymers, and pozzolanic or blended cements [63,64,65,66,67,68,69,70,71,72,73,74,75,76,77,78,79,80,81,82,83,84]. Globally, the industrial-scale conversion of fly ash into artificial aggregates has emerged as a proven strategy. This widespread practice significantly curtails the construction sector’s heavy reliance on virgin aggregates, thereby mitigating the rapid exhaustion of finite natural resources [64,85,86,87]. Moreover, limiting aggregate quarrying and its subsequent high-emission operations not only delivers a marked drop in carbon dioxide levels but also protects coastlines, river basins, and the countryside from destructive environmental impacts [64,86,88]. Furthermore, repurposing FA for aggregate fabrication minimizes the spatial requirements for ash disposal facilities, thereby acting as a crucial safeguard against the contamination of local air quality and groundwater systems [89,90,91]. In current practice, the fabrication of FA aggregates relies primarily on two prevalent methodologies: cold bonding and sintering [85,92,93,94,95,96,97,98,99,100,101]. Relying strictly on the material’s pozzolanic properties, cold bonding serves as a highly economical substitute for the high-energy sintering method. However, this non-thermal approach generally produces structurally weaker aggregate particles [102,103,104]. The mechanical behavior of concrete mixtures incorporating cold-bonded fly ash aggregates has been extensively examined in recent literature [89,94,102,105,106,107]. Substituting conventional normal-weight aggregates with cold-bonded fly ash pellets generally diminishes the mechanical performance of the concrete. Nevertheless, this drawback can be effectively mitigated; mixtures formulated with aggregates that are pre-coated, specifically through surface impregnation using water glass or a cement-silica fume slurry, exhibit noticeably superior mechanical characteristics compared to those containing raw, untreated particles [94,105,108]. Similarly, the mechanical properties of concrete produced with plain cold-bonded fly ash aggregates increased when fly ash aggregates were treated by adding polypropylene fibers and tire chips into fly ash pellets for reinforcing them during agglomeration process [109].

While the aforementioned studies extensively document the isolated use of various recycled aggregates, there remains a significant gap in evaluating fundamentally different waste streams (e.g., concrete, masonry, and fly ash) within a singular, unified comparative framework using full natural aggregate replacement. This study bridges that gap by providing a direct, unbiased mechanical performance comparison and evaluating the reliability of existing code-based prediction models for recycled aggregate concretes.

In this study, the mechanical properties of sustainable concretes produced with different recycled and by-product aggregates were investigated. Recycled concrete aggregates (RCA) and recycled brick aggregates (RBA), representing construction and demolition (C&D) waste streams, together with cold-bonded recycled fly ash aggregates (FAA) produced from thermal power plant fly ash, were used as coarse aggregates by fully replacing natural crushed stone aggregates (CSt) by volume. Six sustainable concrete mixtures were prepared using untreated aggregates (RCA, RBA, FAA) and slag slurry–treated aggregates (TRCA, TRBA, TFAA). Aggregate treatment was carried out using a slag slurry–based two-stage mixing approach (TSMA) incorporating ground granulated blast furnace slag (GGBFS). In addition, microstructural investigations were conducted to evaluate the influence of aggregate treatment on the mechanical performance of recycled aggregate concretes.

Furthermore, the experimental results were compared with estimates obtained from prediction models specified in various design codes, including TS500 (Turkish code) [110], ACI [111,112], Eurocode-2 [113], NZS 3101-1:2006 (New Zealand code) [114], and CSA A23.3-04 (Canadian code) [115]. These models, originally developed for estimating the modulus of elasticity, splitting tensile strength, and modulus of rupture of conventional concrete, were evaluated for their applicability to concretes incorporating recycled and by-product aggregates. The selected codes represent widely used international standards together with national standards from different countries, enabling a comprehensive assessment of model applicability across different design approaches. In addition, alternative regression-based models were developed using the experimental results to improve prediction capability for recycled aggregate concretes.

## 2. Research Significance

This study presents a comprehensive and systematic evaluation of the mechanical performance of recycled aggregate concretes produced using three fundamentally different coarse aggregate types: recycled concrete aggregates (RCA), recycled brick aggregates (RBA), and pelletized recycled fly ash aggregates (FAA). RCA and RBA represent construction and demolition (C&D) waste streams, while FAA is derived from an industrial by-product. By employing identical mix design parameters, curing conditions, and full replacement ratios, the study enables a direct and unbiased comparison of waste-derived and by-product-based aggregates within a unified experimental framework. The consistent use of a slag slurry–based two-stage mixing approach (TSMA) for all aggregate types eliminates confounding effects associated with multiple treatment methods and allows a controlled evaluation of both aggregate type and treatment condition (treated versus untreated).

A key novelty of this work lies in demonstrating the comparative effectiveness of a single, practical, and potentially scalable treatment method applied uniformly across aggregates with distinctly different physical and mechanical characteristics, coupled with a rigorous evaluation of code-based prediction models for these specific concrete mixtures. The proposed TSMA-based approach enhances aggregate quality and interfacial bonding without requiring complex processing steps, making it compatible with conventional concrete production. The results show that slag slurry treatment leads to statistically significant improvements in mechanical properties, while also reducing data variability and enhancing the statistical reliability of recycled aggregate concrete. In this context, the study provides experimentally supported evidence for improving the consistency of concrete produced with heterogeneous waste-derived aggregates.

Another original contribution is the systematic evaluation of widely used international and national code-based prediction models (TS500, ACI, Eurocode-2, NZS 3101-1:2006, CSA A23.3-04) for estimating key mechanical properties, including modulus of elasticity, splitting tensile strength, and modulus of rupture. These models, originally developed for conventional concrete, were assessed for their applicability to concretes incorporating different recycled and by-product aggregates at full replacement levels. The results quantify model accuracy and identify tendencies for underestimation or overestimation depending on aggregate type and treatment condition, thereby clarifying the conditions under which these models can be reliably applied. This is particularly important because assessing the applicability and reliability of existing code-based prediction models for recycled aggregate concretes is essential for their safe and practical implementation in structural design. In addition, alternative regression-based models were developed to improve prediction accuracy.

Overall, this study advances the understanding of recycled aggregate concrete by integrating aggregate type, treatment methodology, mechanical performance, statistical reliability, and code applicability within a single experimental framework. The findings provide both scientific insight and practical guidance for the effective utilization of C&D wastes and industrial by-products in structural concrete, contributing not only to the scientific literature but also to practical design and standardization efforts that support the large-scale adoption of sustainable concrete materials.

## 3. Experimental Study

### 3.1. Materials

CEM I 42.5 R Portland cement was used to produce both FAA and concrete mixtures. F-type fly ash, obtained from Çatalağzı Thermal Power Plant in Zonguldak, Turkey, was utilized in making FAA. Beyond acting as a supplementary cementitious substitute in standard concrete mixtures, GGBFS has also been successfully employed as an effective micro-filler to upgrade the quality of recycled aggregates. The chemical compositions and physical properties of cement, fly ash, and GGBFS are shown in Table 1. Based on these oxide compositions, the fly ash used complies strictly with the requirements of Class F as specified by ASTM C618 [116] (i.e., SiO_2_ + Al_2_O_3_ + Fe_2_O_3_ ≥ 70%, SO_3_ ≤ 5%).

Natural aggregates of river sand and crushed sand (0–4 mm), with specific gravities of 2.65 and 2.70 respectively, were used as fine aggregates in the production of all concrete mixtures. Crushed stone (CSt) virgin coarse aggregates (No. I: 4–8 mm and No. II: 8–16 mm) were used in the control mixture (CStC). Meanwhile, recycled brick aggregates (RBA), recycled concrete aggregates (RCA), and recycled fly ash aggregates (FAA) were used as a complete replacement, by volume, for CSt in the production of both untreated and treated recycled aggregate concrete mixtures: RBAC, RCAC, FAAC, and TRBAC, TRCAC, TFAAC, respectively. This complete replacement was carried out to assess the mechanical performance of each recycled aggregate concrete under the most unfavorable mix proportioning conditions, simulating a worst-case mechanical scenario in which no natural coarse aggregates are utilized.

The crushed RBA and RCA were obtained from an aggregate recycling plant operated by İSTAÇ (Istanbul Environmental Management Industry and Trade Corporation). The aggregate recovery procedure at the facility generally unfolds through a systematic sequence. Initially, raw CDW from the site is broken down into manageable fragments by an excavator fitted with a hydraulic breaker. Following this preliminary size reduction, a track loader feeds the concrete and masonry rubble into a jaw crusher. Subsequent to this primary crushing stage, a magnetic extraction system is employed to remove any embedded steel reinforcement. Ultimately, the processed material is mechanically screened into specific particle size fractions before hauling trucks convey the finished aggregates to the designated stockpiles. It should be noted that there were almost no contaminants, such as tiles, ceramics, wood, metal, plastics, paper, or glass, in the recycled concrete and brick aggregates used in this study, because concrete (either plain or reinforced) and masonry elements were well separated from these impurities at the recycling plant before they were processed. Besides, the RBA used in this study contains hardly any of the mortar fraction and differs from typical crushed brick masonry aggregate, which generally contains mortar to some extent, either attached to aggregates or detached as separate recycled mortar aggregates. This might be attributed to the fact that brick units were separated from the mortar phase as much as possible during the recycling process by the institution supplying the RBA and RCA. Even though it is challenging and generally not feasible to further process recycled aggregates to improve their quality at a recycling plant, due to the limited amount of brick units available in the institution’s storage, they were well separated from the mortar fraction. This separation has possibly been achieved since the masonry demolition waste at the recycling plant is obtained from masonry walls of reinforced concrete buildings, where brick units have a small contact area with mortar relative to the volume of the brick unit within horizontal and vertical joints. Additionally, the mortar-brick bond in joints is usually weak due to technical errors and relatively poor workmanship in older buildings in Turkey. Therefore, crushing the masonry wall pieces of demolished buildings at the recycling plant may yield mostly recycled brick aggregates with no attached mortar.

Recycled fly ash aggregates (FAA) were produced through the cold-bonding agglomeration process under laboratory conditions. As a chemical admixture, Rheobuild-1000, an ASTM C494 [117] Type F high-range water-reducing superplasticizer (SP), was used in concrete mixtures to ensure the required workability of fresh concrete. The properties of the SP are given in Table 2.

### 3.2. Production of Recycled Fly Ash Aggregates

Fly ash aggregates were produced through the cold-bonding agglomeration process using a pelletizing disc with a diameter of 80 cm and a height of 40 cm. To produce fly ash pellets, dry fly ash-cement mixtures were fed into the disc at an inclination angle of 43° and a rotation speed of 45 rpm, which were found by Baykal and Döven [78] to be optimal for this disc size to pelletize fly ash-lime and fly ash-cement mixtures into aggregates with the highest possible unit weight and crushing strength. In the next step, to obtain spherical pellets, water was sprayed onto the powder mixtures during the first 10 min of the agglomeration process, at an amount of 23–27% by weight of the material to be pelletized. An additional 10 min was then allocated for enlarging and further compacting the fresh pellets. Fly ash aggregates were produced with a cement-to-fly ash ratio of 0.1 by weight. To prevent the loss of internal moisture necessary for cement hydration, the fresh pellets were sealed in plastic bags. They were then left to harden inside a climate-controlled curing room at a temperature of 20 ± 2 °C and 90 ± 5% relative humidity (RH) for 28 days to ensure uniform autogenous curing.

### 3.3. Characterization of Coarse Aggregates

**Figure 1:**
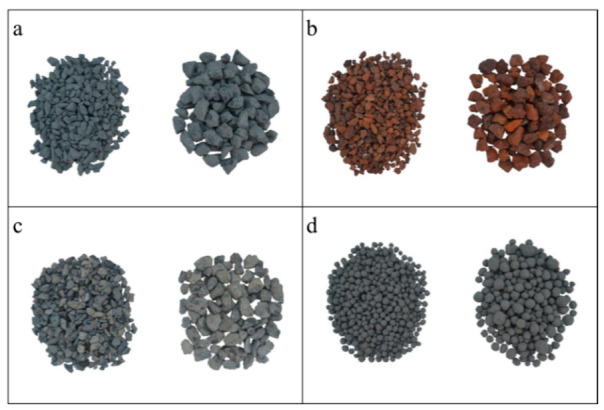
Coarse aggregates separated into different size fractions: (**a**) CSt, (**b**) RBA, (**c**) RCA, (**d**) FAA.

As illustrated in Figure 1, the RBA, RCA, and FAA materials were mechanically graded into 4–8 mm and 8–16 mm fractions to serve as respective alternatives for the No. I and No. II CSt coarse aggregates. The physical characterization of these materials included measuring their unit weight (ASTM C29 [120]), alongside specific gravity and water absorption (ASTM C127 [121]). Additionally, the flakiness index (FI, %) of the coarse fractions was assessed in compliance with the BS EN 933-3 [122] standard. Given the specific conditions under which the recycled aggregates were sourced and processed in this research, evaluating their geometric characteristics, specifically the flakiness index, is imperative. Elevated flakiness values can severely compromise concrete performance by impairing workability, driving up water demand, and trapping excess air, all of which ultimately degrade mechanical strength. To prevent these issues and guarantee adequate batch quality, standard guidelines advise keeping the flakiness index of crushed stone or gravel below the 40% threshold [123]. To explore the relationship between the aggregate characteristics and the ultimate compressive strength of the resulting concrete mixtures, the aggregate crushing values (ACV, %) for the CSt, RCA, RBA, and FAA samples were evaluated in strict accordance with the BS 812-110 [124] standard. To guarantee the formulation of a uniform and well-integrated concrete mixture, the optimum aggregate gradation was designed in compliance with the TS 802 [125] specifications.

### 3.4. Design of Concrete Mixtures

The control concrete (CStC) was produced using Portland cement, GGBFS, river sand, crushed sand, and crushed stone coarse aggregates. Crushed stone No. I and No. II were used in equal volumes, along with water at a water-to-cement ratio (w/c) of 0.50. A sufficient amount of superplasticizer (SP) was added to achieve a slump of 18 ± 2 cm. The detailed mix proportions are presented in Table 3. The optimum mix proportions of coarse aggregates (total of crushed stone No. I and No. II), crushed sand, and river sand were determined to be 55%, 40%, and 5% of the total aggregate volume, respectively. This specific combination ensured that the continuous particle size distribution curve of the combined aggregates fell strictly within the ideal grading boundary limits specified by TS 802 [125]. The maximum grain size of the aggregates used in this study was 16 mm. The total cementitious material in the concrete mixture was 450 kg/m^3^, with a cement replacement ratio for GGBFS determined to be 40% by weight, which is recognized in the literature as the optimum ratio [126]. The water content in the concrete mixture was calculated based on a cement equivalence factor of 0.8 for GGBFS, in accordance with TS 13515 [127] and TS EN 206-1 [128]. A reference concrete was designed to reach an expected compressive strength of 40 to 50 MPa. Based on this control formulation, six alternative mixtures were produced by replacing the standard crushed stone aggregates (No. I and No. II) on a volume basis. For these new batches, the recycled aggregates were integrated either in their untreated form (RBA, RCA, FAA) or conditioned via a GGBFS slurry treatment (TRBA, TRCA, TFAA). RBA, RCA, and FAA were each used as a complete replacement for CSt coarse aggregates in the recycled aggregate concrete mixtures to evaluate the most disadvantageous situation for each type of recycled aggregate.

### 3.5. Casting, Curing, and Testing of Concrete Specimens

In order to minimize slump loss due to the high water absorption of the recycled aggregate, they were first submerged in water for 24 h to ensure saturation and then left on large sieves for 1 h to dry surface before mixing. While these standard immersion and surface drying protocols were strictly followed to establish a consistent saturated surface dry (SSD) baseline, the wide variance in water absorption capacities among these heterogeneous alternative aggregates represents an inherent characteristic of waste-derived materials. To further advance the global understanding of these concrete systems, the precise tracking of localized, micro-level dynamic moisture exchange within the ITZ during the initial mixing phases is recommended as a valuable area for future research using advanced micro-analytical techniques, ensuring that any subtle micro-environmental variations from the global design water-to-binder ratio of 0.50 can be mapped comprehensively.

**Figure 2:**
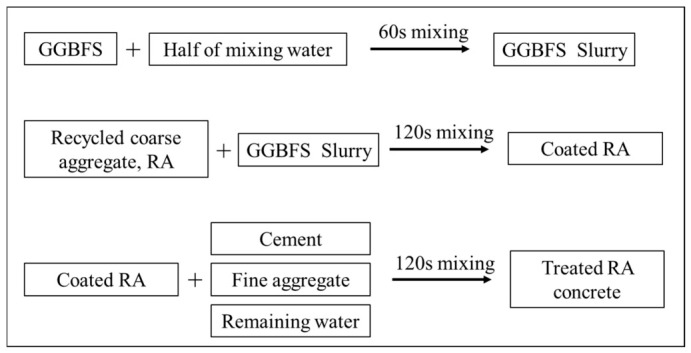
TSMA for producing treated recycled aggregate concrete.

The concrete mixtures of CStC, RCAC, RBAC, and FAAC were produced according to ASTM C192 [129]. However, a TSMA was adopted for casting TRCAC, TRBAC, and TFAAC, as illustrated in Figure 2, with reference to previous studies [51,52,58,130]. For these mixtures, all the GGBFS was first mixed with half of the mixing water for 60 s in a bucket to create the GGBFS slurry using a stirrer motor. The recycled coarse aggregates were then mixed with the GGBFS slurry in a concrete mixer for two minutes. Afterward, the remaining materials were added to the mixer, and an additional two minutes were allowed for mixing to obtain fresh concrete. This approach aimed to fill the voids and cracks on the surface of the recycled aggregates with fine slag grains and to enhance the interface between these aggregates and the cement paste in the concrete through the formation of hydration products resulting from the pozzolanic activity of the slag, both of which increase the microstructural density.

The slump and unit weight of the fresh concrete were measured immediately after production, in accordance with ASTM C143 [131] and ASTM C138 [132], respectively. Following that, concrete specimens were cast and kept in a laboratory environment for 24 h, then demolded and stored in a curing pool at a temperature of 20 ± 2 °C for 28 days until testing.

For each concrete mixture, as shown in Table A1 and Table A2, at least twelve cylindrical specimens of 100 × 200 mm were cast for both the compressive strength and the splitting tensile strength tests, which were performed in accordance with ASTM C39 [133] and ASTM C496 [134], respectively. A test to measure the modulus of elasticity was also performed on the cylinder specimens that were tested for compressive strength, in accordance with ASTM C469 [135]. Additionally, twelve 100 × 100 × 350 mm beam specimens with a 30 mm notch at mid-span were prepared and tested for each concrete mixture to determine the modulus of rupture according to JCI-S-001 [136]. Beam specimens with a clear span of 300 mm were subjected to a three-point bending test under static loading at a constant crack mouth opening displacement (CMOD) rate of 0.05 mm/min. The net modulus of rupture of the beam specimens was calculated using Equation (1) as recommended by ASTM D7264 [137], where *P* is the maximum load (N), *L* is the support span (mm), *b* is the width of the beam specimen (mm), h is the depth or thickness of the beam specimen (mm), and $a_{0}$ is the notch depth (mm).

(1) $$F_{net}=\frac{3PL}{2b{\left(h-a_{0}\right)}^{2}}$$

### 3.6. Statistical Analysis

To assess whether the observed improvement in the mechanical properties of recycled aggregate concretes (RACs) after treatment is statistically significant at a 95% confidence level, a series of statistical tests were conducted. The normality of the data was first evaluated using Skewness, Kurtosis, and the Shapiro-Wilk test. Following this, the assumption of equal variances between treated and untreated concrete groups was examined using the F-Test: Two-Sample for Variances. Based on the results of these tests, the independent *t*-Test: Two-Sample Assuming Equal Variances was applied to determine whether the differences in mechanical properties between treated and untreated RACs were statistically significant.

All statistical analyses were performed using IBM SPSS Statistics Version 20 software and Microsoft Excel’s Data Analysis Tool. The independent *t*-Test: Two-Sample Assuming Equal Variances was applied to each mechanical property for all paired concrete groups, specifically: TRCAC vs. RCAC, TRBAC vs. RBAC, and TFAAC vs. FAAC.

Since the treatment process led to an observed increase in mechanical properties, *p*-values for one-tail were considered to determine whether the improvements were statistically significant. A one-tailed test was specifically selected because the physical mechanism of the TSMA treatment (filling aggregate pores and microcracks with fine slag particles) provides a strict directional expectation that the mechanical properties will either improve or remain unchanged, rather than degrade. Furthermore, because the research objective was strictly to evaluate the effect of the treatment within each specific aggregate type independently (e.g., exclusively comparing RCAC to TRCAC), independent pairwise *t*-tests were deemed the most statistically appropriate method, rendering multi-group omnibus testing (such as ANOVA) unnecessary for the scope of these isolated comparisons. The statistical analysis aimed to verify the following hypotheses:

- Null Hypothesis (H_0_): The mechanical properties of treated RACs are less than or equal to those of untreated RACs. (μ_treated ≤ μ_untreated)
- Alternative Hypothesis (H_a_): The mechanical properties of treated RACs are greater than those of untreated RACs. (μ_treated > μ_untreated)

The statistical significance of the effect of slag slurry treatment was assessed using the *p*-value, also known as the significance probability. The treatment effect was considered statistically significant if *p*-value < 0.05, indicating that the observed improvements in mechanical properties were unlikely to have occurred by random chance. Conversely, if *p*-value > 0.05, the improvements were deemed statistically insignificant.

By conducting these statistical tests, the reliability of the mechanical property enhancements due to the recycled aggregate treatment was rigorously evaluated, ensuring the robustness of the findings presented in this study.

### 3.7. Microstructural Investigation

An environmental scanning electron microscope (ESEM) was used to observe pores, cracks, crystal structures, and morphological changes in the aggregate particles and aggregate-matrix interfaces of both untreated and treated concrete mixtures. Elemental compositions were also examined through energy dispersive X-ray (EDX) analysis.

X-ray diffraction (XRD) analyses were performed on powder samples taken from the aggregate-matrix interfaces in recycled aggregate concrete mixtures on the 28th day to identify changes in the microstructure due to the aggregate slurry treatment process. The samples were obtained from the core region of the concrete elements, rather than from the surface or its vicinity. Qualitative analysis was conducted on the XRD data to determine the crystalline phases and their mineralogical compositions in the samples. Following this, quantitative analysis was carried out to determine the mass percentages of these crystalline phases on the XRD patterns using the Rietveld refinement method with the Material Analysis Using Diffraction (MAUD) software (version 2.9993) [138]. The refinement of the diffraction data was limited to an angular range of 5–40° (2θ), as the most resolved peaks with the highest intensities appeared within this interval. The American Mineralogist Crystal Structure Database was utilized to obtain crystallographic information files. Once the mass percentages of the phases in the samples were determined, the cellular parameters were iterated to minimize errors caused by data shifts and thus to finalize the mass percentages of the phases. Finally, the results were interpreted in light of the phase transformations expected from the pozzolanic reaction.

## 4. Test Results and Discussion

### 4.1. Properties of Coarse Aggregates

The properties of the coarse aggregates used to produce the concrete mixtures in this study are presented in Table 4. FAA is the least dense of all coarse aggregates, followed by RBA, RCA, and CSt. The water absorption capacity of CSt is significantly lower than that of FAA, RBA, and RCA. These trends are consistent with the literature [36,89,139,140,141] and can be attributed to the presence of old adhered mortar on recycled concrete aggregates and the porous structure of recycled brick and fly ash aggregates. Moreover, the water absorption values of recycled aggregates in the size range of 8–16 mm are lower than those of aggregates sized 4–8 mm, while the specific gravity results are higher for the coarser recycled aggregates. Thus, it can be inferred that specific gravity increases while water absorption capacity decreases with an increase in the size of recycled aggregates. The coarser portion of the RCA is expected to be denser due to the lower amount of old adhered mortar, which contains open pores and cracks, resulting in decreased water absorption, as observed by Güneyisi et al. [36]. Additionally, breaking the RCA into smaller pieces damages the aggregate, making it more vulnerable to water absorption, which also applies to RBA. FAAs also became denser with fewer, mostly non-interconnected closed pores when the pellets increased in size during agglomeration, leading to decreased water absorption and increased specific gravity, as noted by Kockal and Ozturan [102] for cold-bonded FAAs. On the other hand, the specific gravity and water absorption capacity of the recycled fly ash aggregates produced in this study fall within a similar range to those reported in the literature [94,105,106,108,142,143].

In terms of the flakiness index, it can be stated that all coarse aggregates are suitable for concrete production according to BS 882 [123], as the FI values of these aggregates are significantly lower than 40%.

**Figure 3:**
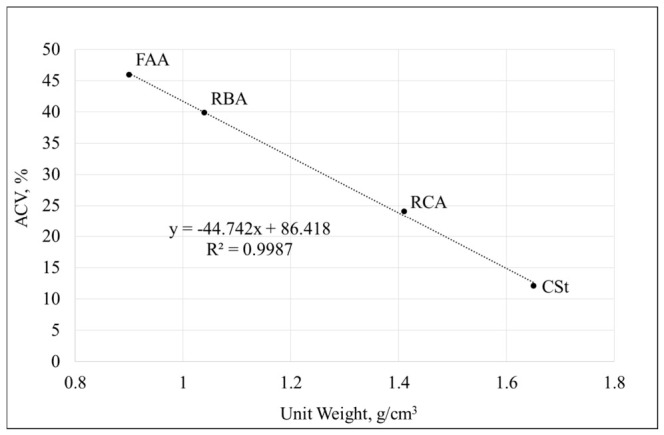
Relationship between aggregate crushing value (ACV, %) and aggregate unit weight.

Regarding ACV, there is a substantial difference among the aggregates, favoring CSt, which has an ACV of 12.1%. This indicates that FAA is the least favorable in terms of crushing strength, as higher ACV values signify more mechanically flawed materials. Furthermore, as expected, there is a negative correlation between aggregate crushing values and the unit weight of aggregates, as presented in Figure 3. These results are also consistent with the literature [56,107,144,145,146,147,148]. The regression model constructed and shown in this figure exhibits high coefficients of determination (R^2^) that highlight the significance of the linear relationship established between the related variables. In other words, as a statistical measure, the coefficient of determination (R^2^) indicates the percentage of variance in the dependent variable that the regression equation can explain [149,150].

### 4.2. Fresh Concrete Properties

Concrete mixtures were produced by employing a superplasticizer (SP) at the required dosage to achieve the target workability. It was observed that FAAC required the least amount of SP to maintain the specified slump value (18 ± 2 cm) owing to the smoother surface texture and the greater roundness of the fly ash aggregates (FAA), which had the lowest flakiness index. On the other hand, reaching the desired slump necessitated the highest superplasticizer (SP) dosage for the CStC mixture (0.85% by binder mass), with RCAC (0.75%) and RBAC (0.65%) requiring progressively less. This trend directly correlates with aggregate morphology; CSt exhibits the most pronounced flakiness and least roundness, followed sequentially by RCA and RBA. Because highly flaky particles possess a larger surface area-to-volume ratio, they inherently demand more fluid to ensure adequate flowability. Ultimately, all the fresh batches demonstrated excellent cohesion and workability without any signs of segregation.

When RCA, RBA, and FAA fully replaced the CSt, the fresh unit weight of the concrete mixtures decreased from 2410 kg/m^3^ to 2260, 2120, and 2000 kg/m^3^, respectively. The use of coarse aggregates with different densities provided the opportunity to produce concrete mixtures with varying unit weights. Alterations in the manufacturing process did not impact the fresh unit weights recorded for TRCAC, TRBAC, and TFAAC. Furthermore, while the complete replacement of traditional crushed stone with recycled alternatives yielded up to a 17% reduction in fresh density, the resulting compressive strengths still satisfied structural requirements. Their compressive strengths successfully met the regulatory limits of 17 MPa (ACI 318-02 [151]) and 21 MPa (ACI 213R-03 [152], which governs structural lightweight-aggregate concrete).

### 4.3. Hardened Concrete Properties

#### 4.3.1. Compressive Strength and Modulus of Elasticity

**Figure 4:**
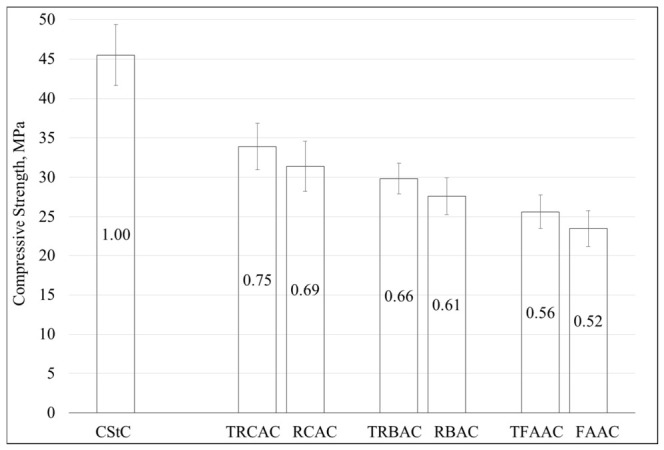
Compressive strength of concretes.

**Figure 5:**
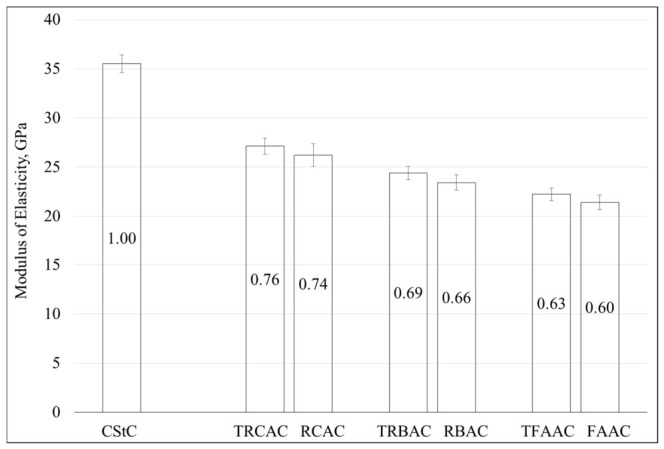
Elastic modulus of concretes.

As detailed in Table A1, the inherently elevated porosity and inferior mechanical capacity of the recycled particles caused a notable decline in the overall performance of the mixtures. Specifically, the compressive strength dropped from 45.5 MPa in the reference batch (CStC) to a range of 33.9 to 23.5 MPa for the recycled aggregate concretes. Within the group of recycled aggregate mixtures, the peak compressive strength was recorded for TRCAC, sequentially followed by RCAC, TRBAC, RBAC, TFAAC, and FAAC, with performances of 75%, 69%, 66%, 61%, 56%, and 52% of CStC, respectively, as seen in Figure 4. Likewise, the data summarized in Table A2 reveals a corresponding drop in the elastic modulus, which shifted from the CStC’s baseline of 35.5 GPa to between 27.1 and 21.4 GPa across the recycled aggregate concretes. The peak modulus of elasticity was observed in the TRCAC batch, ranking ahead of RCAC, TRBAC, RBAC, TFAAC, and FAAC, with performances of 76%, 74%, 69%, 66%, 63%, and 60% of CStC, respectively, as seen in Figure 5. The improvements due to treating recycled aggregates with GGBFS slurry can be attributed to the combined effects of filling voids and microcracks on the aggregate surface with fine slag particles, and the formation of hydration products from the pozzolanic activity of the slag. Together, these processes increase microstructural density, strengthening the interface between the recycled aggregates and the concrete matrix. It is critical to note that the effectiveness of the TSMA is fundamentally rooted in the spatial redistribution of the cementitious materials and water. By premixing the aggregates with the GGBFS slurry, the treatment deliberately enriches the highly porous Interfacial Transition Zone (ITZ) with GGBFS particles. Therefore, the observed performance improvements are not solely due to a generic surface modification, but are directly driven by this targeted spatial redistribution, which concentrates pozzolanic activity exactly where the microstructural defects are most prevalent. Consistent with these findings, several previous studies [53,54,55,57,58,153] have reported comparable enhancements in both compressive strength and elastic modulus when recycled aggregates are treated with a pozzolanic slurry during the batching process.

To accurately gauge statistical consistency, the coefficient of variation (CoV) is utilized as a practical measure of dispersion, given that it takes both the mean and the standard deviation into consideration. From this point view, while the CoV values of the concretes RCAC, RBAC, and FAAC were 10.1%, 8.5%, and 9.8% for compressive strength, and 4.4%, 3.4%, and 3.5% for modulus of elasticity, they decreased to 8.7%, 6.5%, and 8.5% for compressive strength, and 3.0%, 2.7%, and 3.0% for modulus of elasticity for the concretes of TRCAC, TRBAC, and TFAAC, respectively. It should be noted that a lower CoV indicates higher statistical reliability [149,150].

To further assess the statistical significance of the observed improvements in the compressive strength and modulus of elasticity, an independent *t*-test (assuming equal variances) was conducted for each pair of untreated and treated recycled aggregate concretes. The results demonstrated that the enhancements observed for the treated concretes were statistically significant, with *p*-values consistently below 0.05, confirming the reliability of the improvements.

For compressive strength, statistical analysis revealed that treating the recycled aggregates led to a significant increase in strength across all concrete types. Specifically, the *p*-values were 0.022 for RCAC vs. TRCAC, 0.006 for RBAC vs. TRBAC, and 0.012 for FAAC vs. TFAAC, all below the 0.05 threshold. These results indicate that the improvements in compressive strength due to treatment were not only observable but also statistically meaningful.

Similarly, for modulus of elasticity, the statistical tests confirmed that the increases were significant, with *p*-values of 0.011 for RCAC vs. TRCAC, 0.001 for RBAC vs. TRBAC, and 0.004 for FAAC vs. TFAAC. These findings validate that the slag slurry treatment had a meaningful impact on enhancing the stiffness of the recycled aggregate concretes, as measured by the modulus of elasticity.

Thus, the statistical evaluation strongly supports the conclusion that the slag slurry treatment had a statistically significant effect on both the compressive strength and modulus of elasticity of the recycled aggregate concretes, reinforcing the structural benefits of this approach.

**Figure 6:**
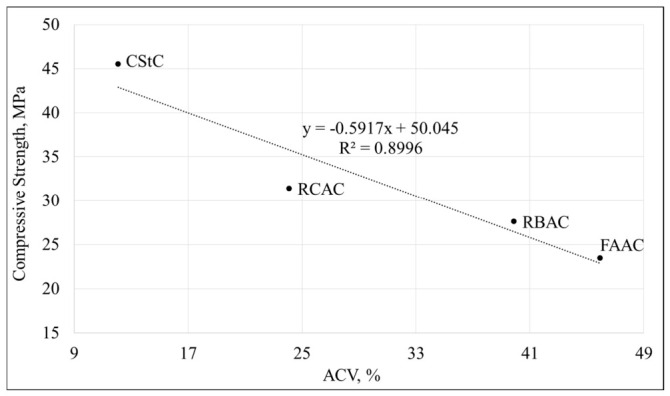
Relationship between compressive strength of concretes and ACV (%) of coarse aggregates.

**Figure 7:**
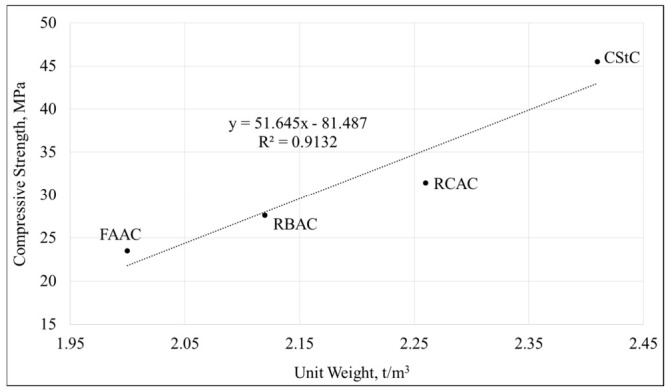
Relationship between unit weight and compressive strength of untreated concretes.

**Figure 8:**
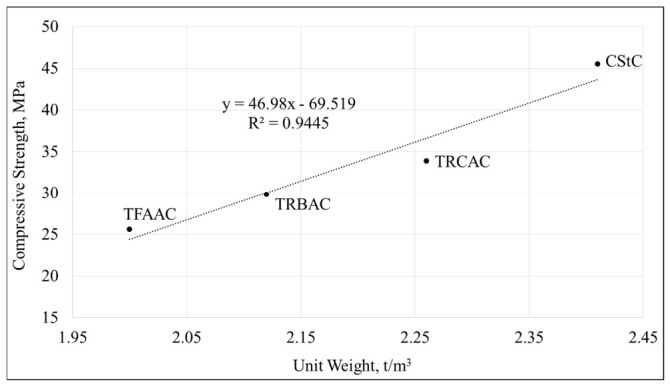
Relationship between unit weight and compressive strength of treated concretes.

On the other hand, as expected, the correlation between the compressive strength of the concretes and the aggregate crushing values (ACV, %) of the coarse aggregates was negative, while it was positive with the unit weight of both untreated and treated concrete mixtures, as shown in Figure 6 and Figure 7 and Figure 8, respectively. All relationships had sufficiently high coefficients of determination (R^2^). However, the relationship between compressive strength and unit weight for the set of concretes including CStC, TRCAC, TRBAC, and TFAAC (R^2^ = 0.9445) was slightly stronger than that for the set of concretes of CStC, RCAC, RBAC, and FAAC (R^2^ = 0.9132).

#### 4.3.2. Splitting Tensile Strength

**Figure 9:**
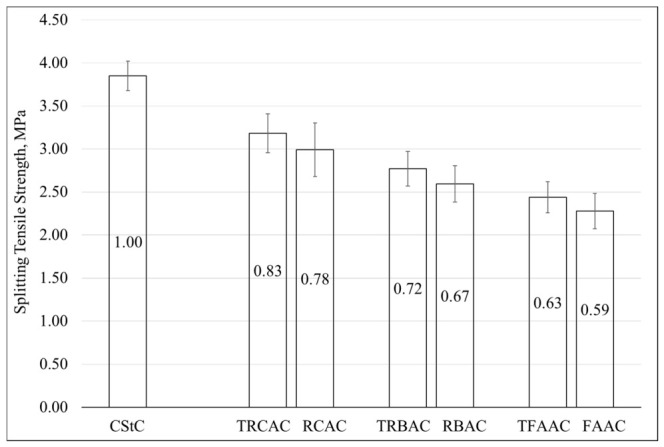
Splitting tensile strength of concretes.

The splitting tensile strength of the concretes decreased from 3.9 MPa for the control concrete (CStC) to a range between 3.2 and 2.3 MPa when crushed stone was replaced with recycled coarse aggregates, as presented in Table A3. Among the recycled aggregate concretes, higher splitting tensile strength was observed in TRCAC, followed by RCAC, TRBAC, RBAC, TFAAC, and FAAC, achieving 83%, 78%, 72%, 67%, 63%, and 59% of the strength of CStC, respectively, as seen in Figure 9. The treatment of recycled aggregates with GGBFS slurry during concrete mixing improved the splitting tensile strength of the recycled aggregate concretes. Previous studies by Güneyisi et al. [36], Tam and Tam [51], and Kisku et al. [55] also observed an increase in the splitting tensile strength of recycled aggregate concrete when the recycled aggregates were treated with pozzolanic admixtures during the multi-step mixing process.

On the other hand, the CoV values of RCAC, RBAC, and FAAC, produced with untreated recycled coarse aggregates, were reduced from 10.4%, 8.2%, and 9.0%, respectively, to 7.1%, 7.3%, and 7.4% for TRCAC, TRBAC, and TFAAC with treated recycled coarse aggregates. The lower the CoV, the higher the statistical reliability. Besides, the statistical analysis performed for each pair of untreated and treated recycled aggregate concretes confirms that the treatment process led to a statistically significant increase in splitting tensile strength, as indicated by *p*-values below 0.05 for all concrete groups.

Specifically, the *p*-values were 0.043 for RCAC vs. TRCAC, 0.022 for RBAC vs. TRBAC, and 0.023 for FAAC vs. TFAAC, demonstrating that the improvements in splitting tensile strength following the slag slurry treatment were statistically meaningful. This finding suggests that reducing microstructural weaknesses within the recycled aggregates and enhancing the interfacial transition zone bond contributed to the increased tensile performance of the treated concrete.

#### 4.3.3. Modulus of Rupture

**Figure 10:**
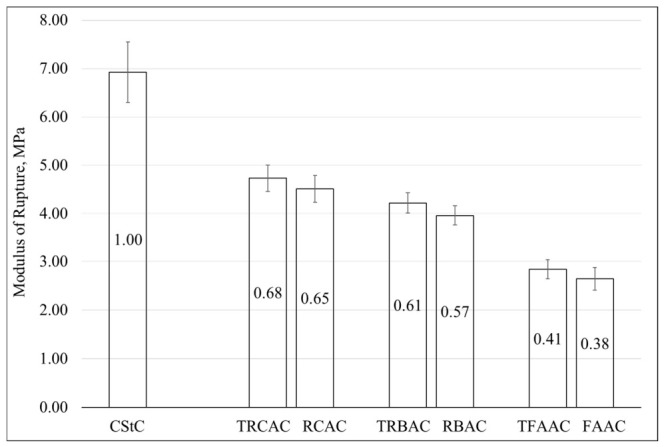
Modulus of rupture of concretes.

The modulus of rupture of the concretes decreased from 6.9 MPa for the control concrete (CStC) to a range between 4.7 and 2.7 MPa when crushed stone was fully replaced with recycled coarse aggregates, as seen in Table A4. Among the recycled aggregate concretes, the highest modulus of rupture was recorded by TRCAC, followed by RCAC, TRBAC, RBAC, TFAAC, and FAAC, achieving performance levels of 68%, 65%, 61%, 57%, 41%, and 38% of CStC, respectively, as shown in Figure 10. The modulus of rupture for the recycled aggregate concretes improved when the recycled aggregates were treated with GGBFS slurry during the production process. Similar trends were observed in previous studies [39,51,58,153], where the modulus of rupture for concrete incorporating recycled aggregates increased when these aggregates were coated with pozzolanic slurry during the mixing stage.

Additionally, the CoV values for RCAC, RBAC, and FAAC, produced with untreated recycled coarse aggregates, decreased from 6.2%, 5.2%, and 8.7%, respectively, to 5.8%, 5.0%, and 6.9% for TRCAC, TRBAC, and TFAAC with treated recycled aggregates. The treatment of recycled aggregates reduced the CoV values, leading to greater accuracy and reliability in the measurements. Moreover, the results of statistical analysis indicate that slag slurry treatment had a significant impact on the modulus of rupture, as reflected in the *p*-values, all of which were below 0.05. This confirms that the observed improvements in modulus of rupture were not due to random variation but were statistically significant.

In particular, the *p*-values for RCAC vs. TRCAC, RBAC vs. TRBAC, and FAAC vs. TFAAC were 0.029, 0.003, and 0.017, respectively. These findings strongly suggest that the treatment process effectively enhanced the modulus of rupture across all concrete groups, reinforcing its role in improving the structural integrity of concrete made with recycled aggregates. The improvements can be attributed to the densification of the interfacial transition zone and the refinement of microstructural defects within the recycled aggregates, both of which contributed to increased resistance against flexural stresses.

#### 4.3.4. Microstructural Findings

**Figure 11:**
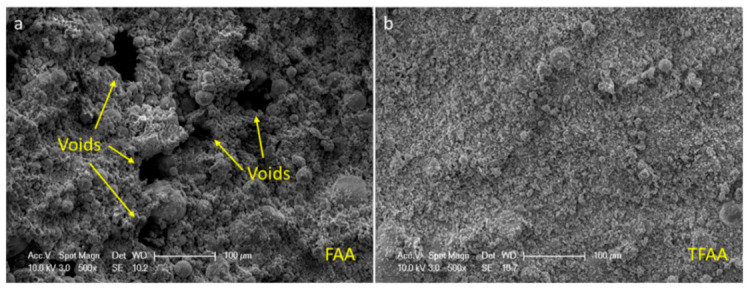
SEM observations: (**a**) FAAC, (**b**) TFAAC.

**Figure 12:**
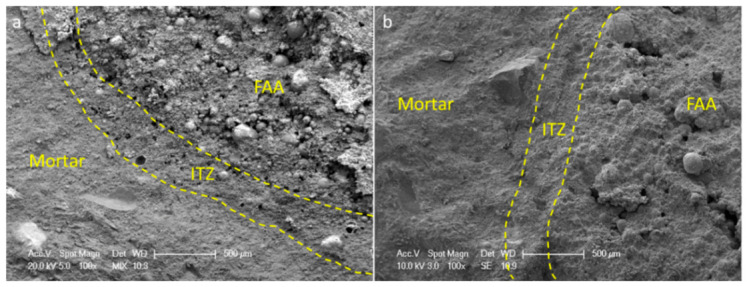
SEM observations: (**a**) FAAC, (**b**) TFAAC.

**Figure 13:**
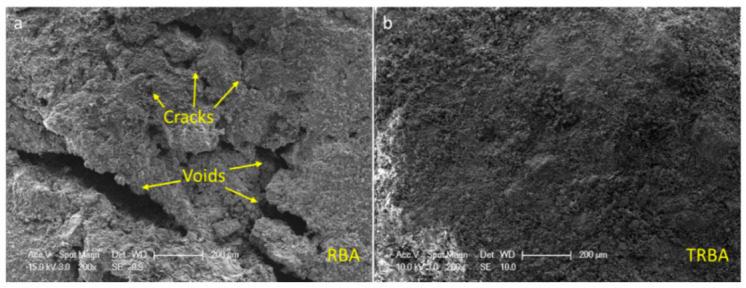
SEM observations: (**a**) RBAC, (**b**) TRBAC.

**Figure 14:**
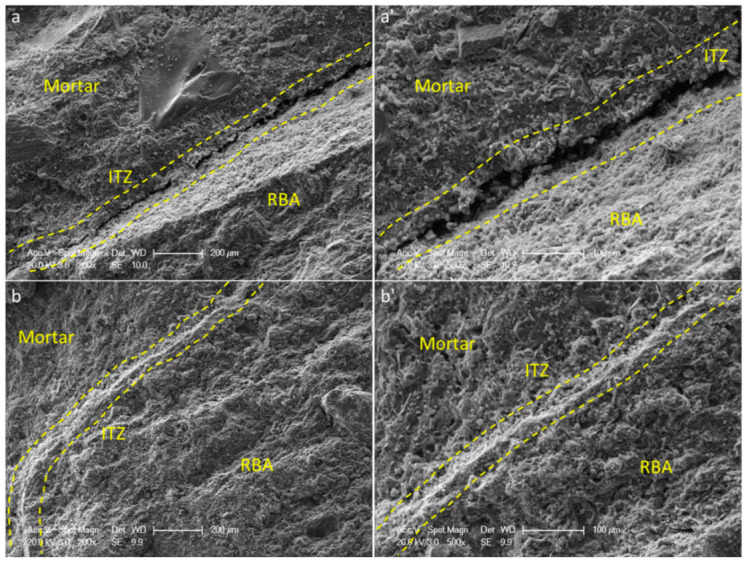
SEM observations: (**a**) RBAC, (**a′**) Zoomed RBAC, (**b**) TRBAC, (**b′**) Zoomed TRBAC.

**Figure 15:**
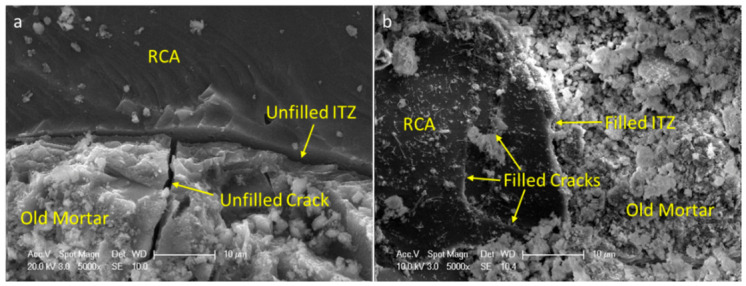
SEM observations: (**a**) RCAC, (**b**) TRCAC.

**Figure 16:**
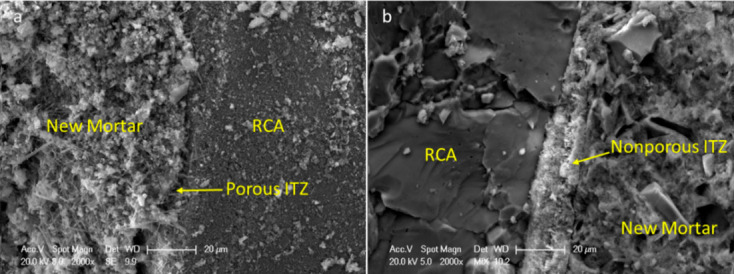
SEM observations: (**a**) RCAC, (**b**) TRCAC.

The effect of the TSMA treatment applied to the recycled aggregates was examined based on the microscopic observations shown in Figure 11, Figure 12, Figure 13, Figure 14, Figure 15 and Figure 16. To prevent the introduction of artificial damage, it is crucial to note that the ESEM analysis was conducted on solid, intact fracture surface fragments obtained directly from the split cylinder specimens after the splitting tensile test, without any grinding or crushing. Figure 11 and Figure 13 display top-view images of aggregates located on these unaltered fracture surfaces of split cylinder specimens. In one half of each specimen, these aggregates detached from the interface, while in the other half, they were still attached before the experiments. In concrete mixtures produced with treated recycled aggregates, slag particles (with a high Blaine fineness of 5253 cm^2^/g) fill the voids and cracks on the surfaces of the aggregates as well as the interfaces between the aggregates and the mortar. This mechanism, achieved by coating the recycled aggregates with slag slurry during the mixing procedure, might have also contributed to the further formation of C-S-H due to the improved efficiency of pozzolanic reactions owing to the higher slag ratio, thereby ensuring that interfaces, voids, and cracks evolved into a denser structural form. As noted in previous studies [154,155,156,157,158], C-S-H gel formations appear in regions with atomic Ca/Si ratios ranging from 0.8 to 2.5. The filled, nonporous regions shown in the aforementioned figures, taken from samples of treated concretes, exhibited a Ca/Si ratio within the range specified in the literature.

**Figure 17:**
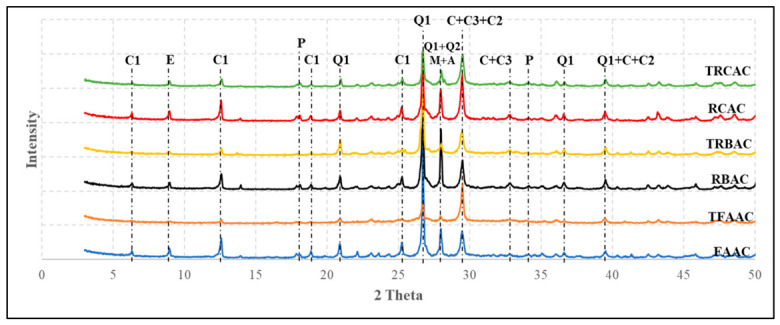
XRD patterns of samples.

As a result of the XRD analysis, the crystallographic phases present in the samples and their compositions, along with the corresponding database codes, are provided in Table 5. These phases, their angles, and sample matches are illustrated in Figure 17. The mass percentages of the crystalline phases present in the samples are shown in Table 6. The powder samples taken from the interfaces consist of fine aggregates used in concrete production and the cement matrix. However, most of the phases in the samples originated from the aggregates. Considering this, any changes in the matrix should be taken into account to understand the effect of the treatment process on the microstructure. For this reason, the matrix was considered as a whole, and Table 7 was obtained by maintaining the ratios between the phases (portlandite, ettringite, and others) that constitute the matrix. Because the raw Rietveld data (Table 6) represents the bulk composition, including highly variable quantities of inert crystalline aggregate phases such as quartz, direct comparison of raw hydration products between different mixtures can be misleading due to aggregate dilution effects. To accurately evaluate pozzolanic activity, the data was matrix-normalized (Table 7). This mathematical normalization isolates the reactive cementitious paste by removing the inert aggregate phases, providing a true comparative reflection of Portlandite consumption and hydration kinetics.

**Table 5:** Crystallographic phases and compositions contained in the samples.

Phases	Abbreviations	Compositions	Database Codes *
Quartz (1)	Q1	SiO_2_	0006212
Quartz (2)	Q2	SiO_2_	0015465
Calcite	C	CaCO_3_	0000984
Clinochlore	C1	Mg_6_Al_4_O_10_(OH)_8_	0002740
Albite	A	NaAlSi_3_O_8_	0001285
Margarite	M	CaAl_2_Si_2_Al_2_O_10_(OH)_2_	9000604
Portlandite	P	Ca(OH)_2_	0000117
Ettringite	E	Ca_6_(Al(OH)_6_)_2_(SO_4_)_3_(H_2_O)_26_	0017886
Belite	C2	Ca_2_SiO_4_	0020214
Alite	C3	Ca_3_SiO_5_	1540704

* The American Mineralogist Crystal Structure Database.

**Table 6:** Phases (mass %) in the samples determined by the Rietveld refinement method.

Concrete Mixtures	Aggregate						Matrix		
	Quartz-1	Quartz-2	Calcite	Clinochlore	Albite	Margarite	Portlandite	Ettringite	Others
FAAC	29.7	0.5	19.3	15.0	20.2	3.1	2.2	1.6	8.3
TFAAC	13.7	1.1	36.6	5.9	24.3	4.5	1.2	0.1	12.7
RBAC	31.6	5.8	19.6	13.2	15.3	3.6	2.4	0.6	7.9
TRBAC	31.6	0.3	24.9	7.3	19.0	5.2	0.3	0.1	11.3
RCAC	25.4	1.5	32.3	9.1	17.1	3.3	2.5	1.0	8.0
TRCAC	17.5	2.4	28.3	7.5	16.0	5.1	3.1	0.8	19.5

**Table 7:** Relative weight percentage by mass calculated for portlandite and ettringite in matrix.

Concrete Mixtures	Matrix	
	Portlandite	Ettringite
FAAC	18.3	12.8
TFAAC	8.3	0.7
RBAC	21.9	5.6
TRBAC	2.6	0.9
RCAC	21.5	8.6
TRCAC	13.2	3.3

In this study, it was assumed that the calcite phase originated exclusively from the aggregate. This assumption was supported by several factors: the samples were cured in a water pool (carbonation proceeds significantly slower in aqueous environments [159]), the analyses were performed after 28 days (a period too short for significant carbonation), and the samples selected for analysis were taken from the core region of the concrete specimens. Therefore, while trace amounts of carbonation might theoretically occur during final sample grinding and handling, its contribution to the total detected calcite content is deemed practically negligible and does not alter the fundamental phase distributions or the subsequent mechanistic interpretations. It was observed that the ettringite and portlandite contents decreased when the recycled aggregates were treated. While the untreated samples also contained 40% slag by weight of cement, this proportion exceeded 40% in the interfacial transition zone (ITZ) as a result of the treatment method. As this ratio increased, the cement content in the ITZ decreased, leading to a reduction in the ettringite and portlandite phases. The increased efficiency of pozzolanic reactions, driven by the higher slag ratio, was also considered responsible for the further reduction in portlandite content. Although XRD primarily identifies crystalline phases, the quantitative depletion of crystalline portlandite (CH) observed in the Rietveld data, coupled with the microstructural densification visible in the SEM images, strongly infers the formation of additional amorphous C-S-H gels (secondary hydration products). This combined mechanism explains the observed decrease in void volume and the subsequent improvement in mechanical strength.

Taken together, the findings presented so far demonstrate that while the improvements in the mechanical properties of recycled aggregate concretes treated with a multi-step mixing technique are not substantial, the gains achieved are nevertheless remarkable, especially given that they were attained operationally by simply modifying the concrete mixing process. These results demonstrate that concretes incorporating recycled aggregates derived from construction and demolition (C&D) waste streams (RCA and RBA) and an industrial by-product (FAA), particularly when treated with slag slurry, can provide a viable alternative for large-scale, environmentally responsible structural applications. This is further supported by the observation that, even with the complete replacement of natural crushed stone coarse aggregates, the compressive strength of these mixtures remains within the limits required for structural use.

In addition, the use of ground granulated blast furnace slag (GGBFS), another industrial by-product, both as a cement replacement material at relatively high levels and as a treatment agent for recycled aggregates, enhances the overall performance of the system. This dual utilization contributes not only to improved mechanical behavior but also to the effective valorization of both C&D wastes and industrial by-products within concrete production.

However, despite these promising outcomes, the durability performance of recycled aggregate concrete should be carefully evaluated in practical applications.

Accordingly, evaluating the applicability and reliability of existing code-based prediction models for such concretes is essential to support their safe and practical implementation in structural design.

### 4.4. Analysis of the Mechanical Properties in Comparison with the Different Prediction Models

In this study, the measured mechanical properties of concretes were compared with those calculated based on prediction models provided in various codes, including TS500 (Turkish code), ACI, Eurocode-2, CSA A23.3-04 (Canadian code), and NZS 3101-1:2006 (New Zealand code). This comparison was conducted to assess the applicability of these models, which are typically used to estimate mechanical properties such as modulus of elasticity, splitting tensile strength, and modulus of rupture in conventional concrete, for recycled aggregate concretes. Moreover, new models, formulated through linear regression analysis, were introduced based on the test results. The codes selected include two widely accepted international standards (ACI and Eurocode-2), as well as additional standards from other Anglo-Saxon countries (New Zealand and Canada) and the national standard of Turkey (TS500), where this study was conducted.

Different models recommended by the specified codes for predicting the modulus of elasticity, splitting tensile strength, and modulus of rupture of concrete are presented in Table 8. To estimate the same property across all models, certain adjustments to some models are required. Eurocode-2 calculates direct tensile strength. For converting direct tensile strength to indirect tensile strength, it was assumed that direct tensile strength equals 90% of the indirect tensile strength [113]. Additionally, in the case of Eurocode-2, the characteristic compressive strength, *f_ck_*, was transformed into the mean compressive strength, *f_cm_*, using the following formula [113]:

(2) $$f_{ck}=f_{cm}-8\;MPa$$

In the Eurocode-2 model, the modulus of rupture is specified based on the mean tensile strength, *f_ctm_*, and the height of the beam specimens. The mean tensile strength is converted to mean compressive strength through the following equation [113]:

(3) $$\;f_{ctm}=0.3{\left(f_{cm}-8\;MPa\right)}^{2/3}$$

The effective height of the beam specimens (70 mm) was taken into account and substituted into the original equation to calculate the modulus of rupture, as per Eurocode-2, based on the compressive strength of the concrete specimens produced in this study.

In the case of prediction models provided by ACI 318-08, ACI 363R-10, CSA A23.3-04, and NZS 3101-1:2006, the characteristic compressive strength, $f_{c}^{\prime }$, was converted to the mean compressive strength, $f_{cr}^{\prime }$, using Equation (4) [111], and then substituted into the equations given in Table 8 to calculate the mechanical properties.

(4) $$\;f_{c}^{\prime }=\left\{\begin{array}{l} f_{cr}^{\prime }-7\;\mathrm{MPa},\;\;\;\;\;\;\;\;\;\;\;\;\;\;\;\;\;\;\;\;\;\;\;\;f_{c}^{\prime }<21\;\mathrm{MPa}\\ f_{cr}^{\prime }-8.5\;\mathrm{MPa},\;\;\;\;\;\;\;\;\;\;\;\;\;\;\;\;\;\;\;\;{21\;MPa\leq f}_{c}^{\prime }\leq 35\;\mathrm{MPa}\\ {(f}_{cr}^{\prime }-5\;\mathrm{MPa})/1.10,\;\;\;\;\;\;\;\;\;\;\;f_{c}^{\prime }>35\;\mathrm{MPa} \end{array}\right.$$

For the prediction models provided by TS500, the characteristic compressive strength, *f_ck_*, was adjusted to the mean compressive strength, *f_cm_*, using the following formula [110]:

(5) $$f_{ck}=f_{cm}-1.28\sigma$$

where σ represents the standard deviation of the compressive strength of concrete samples tested.

In this study, the comparisons between the experimental means of the mechanical properties investigated and the predictions provided by various codes for the relevant mechanical properties were categorized based on the following criteria:

- For differences equal to or less than 1%, the discrepancy is categorized as negligible overestimation or negligible underestimation.
- Differences exceeding 1% but equal to or less than 5% are considered slight overestimation or slight underestimation.
- Differences exceeding 5% but equal to or less than 15% represent moderate overestimation or moderate underestimation, while differences exceeding 15% are categorized as significant overestimation or significant underestimation.

Although the standard deviations of the mechanical properties investigated in this study reach up to 10% relative to their mean values, the smaller intervals specified above were used as a reference for comparisons. This approach emphasizes the predictive capabilities of the models more clearly and highlights their applicability to concretes produced with recycled aggregates, despite being originally developed for conventional concrete.

#### 4.4.1. Modulus of Elasticity

**Figure 18:**
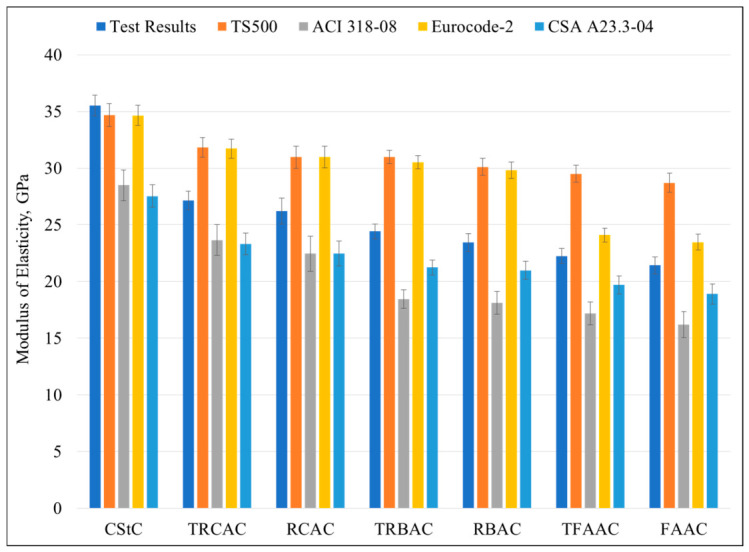
Measured and predicted results for modulus of elasticity.

As shown in Table A2 and Figure 18, the mean modulus of elasticity values for TS500 and Eurocode-2 were very close to the experimental mean for the control concrete (CStC), with values of 34.7, 34.6, and 35.5 GPa, respectively. In contrast, the models of ACI 318-08 and CSA A23.3-04 significantly underestimated the modulus of elasticity; yielding values of 28.5 and 27.5 GPa, respectively.

In the case of concrete mixtures produced with recycled concrete aggregates, TS500 and Eurocode-2 significantly overestimated the modulus of elasticity by 18.2% and 18.3% for RCAC, and by 17.3% and 16.9% for TRCAC, respectively. However, the ACI 318-08 and CSA A23.3-04 models showed moderate underestimations of 12.8% and 14.1% for TRCAC, respectively, and approximately 14.3% for RCAC in both models.

For the concrete mixtures incorporating recycled brick aggregates, the TS500 and Eurocode-2 models demonstrated significant overestimations, with values of 28.5% and 27.2% for RBAC, and 27.0% and 25.1% for TRBAC, respectively. In contrast, the ACI 318-08 and CSA A23.3-04 models showed a significant underestimation of 22.7% and a moderate underestimation of 10.5% for RBAC, respectively. For TRBAC, these models indicated a significant underestimation of 24.5% and a moderate underestimation of 13.0%, respectively.

For concrete mixtures utilizing recycled fly ash aggregates, the TS500 model exhibited significant overestimations of 33.9% and 32.7%, while the Eurocode-2 model demonstrated moderate overestimations of 9.5% and 8.3% for FAAC and TFAAC, respectively. Conversely, the ACI 318-08 model revealed significant underestimations of 24.5% and 22.7%, whereas the CSA A23.3-04 model reflected moderate underestimations of 11.8% and 11.5%, for FAAC and TFAAC, respectively.

#### 4.4.2. Splitting Tensile Strength

**Figure 19:**
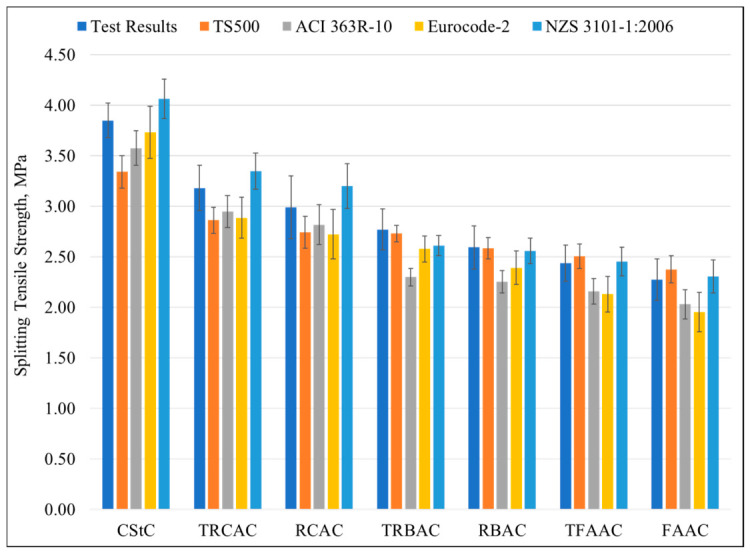
Measured and predicted results for splitting tensile strength.

As presented in Table A3 and Figure 19, the mean splitting tensile strength value predicted by Eurocode-2 closely aligns with the experimental mean for CStC, with values of 3.7 MPa and 3.9 MPa, respectively. In addition, the NZS 3101-1:2006 model demonstrated a moderate overestimation of 5.5% for CStC. By contrast, the TS500 and ACI 363R-10 models exhibited moderate underestimations of 13.2% and 7.1%, respectively.

For the recycled concrete aggregate mixtures, the NZS 3101-1:2006 model demonstrated moderate overestimations of 7.0% and 5.3% for RCAC and TRCAC, respectively. In contrast, the TS500, ACI 363R-10, and Eurocode-2 models exhibited moderate underestimations for both mixtures. For RCAC, these models predicted values below the experimental mean by 8.3%, 5.8%, and 8.9%, respectively, while for TRCAC, the corresponding deviations were 10.0%, 7.3%, and 9.2%.

For concrete mixtures with recycled brick aggregates, the TS500 and NZS 3101-1:2006 models produced mean splitting tensile strength values that closely aligned with the experimental means, showing negligible underestimation of 0.4% and slight underestimation of 1.3% for RBAC, respectively. Similarly, the TS500 model demonstrated close agreement with the experimental mean for TRBAC, with a slight underestimation of 1.4%. The ACI 363R-10 and Eurocode-2 models showed moderate underestimations for RBAC, with deviations of 13.1% and 7.8%, respectively. For TRBAC, the ACI 363R-10 model exhibited a significant underestimation of 17.0%, while the Eurocode-2 and NZS 3101-1:2006 models demonstrated moderate underestimations, with discrepancies of 6.9% and 5.8%, respectively.

For concrete mixtures produced with recycled fly ash aggregates, the mean splitting tensile strength values predicted by the NZS 3101-1:2006 model closely aligned with the experimental means, showing a slight overestimation of 1.3% for FAAC and a negligible overestimation of 0.7% for TFAAC. The TS500 model exhibited slight overestimations of 4.4% for FAAC and 2.8% for TFAAC. In contrast, the ACI 363R-10 and Eurocode-2 models demonstrated moderate underestimations for both FAAC and TFAAC, with deviations of 10.8% and 14.2% for FAAC, and 11.4% and 12.6% for TFAAC, respectively.

#### 4.4.3. Modulus of Rupture

It is imperative to note that the prediction models provided by ACI, Eurocode, CSA, and TS500 are empirically derived for the flexural tensile strength of standard, unnotched concrete beams. In this study, testing was conducted on notched beams to evaluate fracture behavior. Therefore, the net modulus of rupture calculated here includes severe stress concentration effects at the notch tip. The comparison presented in this section is not intended to validate the code equations, but rather to use the unnotched code predictions as a theoretical upper-bound baseline to quantify the notch-sensitivity and defect-tolerance of the treated recycled aggregate concretes.

**Figure 20:**
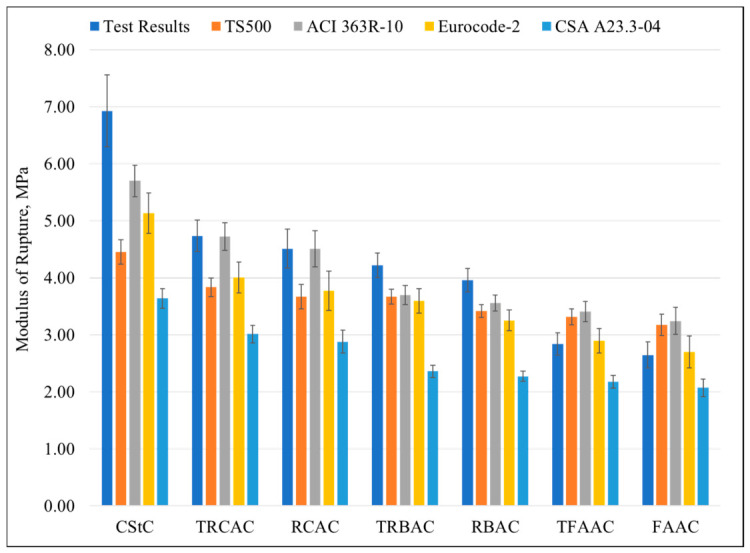
Measured and predicted results for modulus of rupture.

As shown in Table A4 and Figure 20, all the mean modulus of rupture values predicted by the code models significantly underestimated the experimental mean for CStC, with deviations of at least 17.7%, as estimated by ACI 363R-10.

For concrete mixtures made with recycled concrete aggregates, the ACI 363R-10 model showed a negligible overestimation for RCAC and a negligible underestimation for TRCAC, with values of 0.01% and 0.2%, respectively. However, the TS500, Eurocode-2, and CSA A23.3-04 models exhibited significant underestimations for both mixtures. For RCAC, the discrepancies were 18.6%, 16.4%, and 36.2%, respectively, while for TRCAC, the corresponding underestimations were 19.0%, 15.4%, and 36.3%, respectively.

For concrete mixtures including recycled brick aggregates, the TS500, ACI 363R-10, Eurocode-2, and CSA A23.3-04 models demonstrated discrepancies for both RBAC and TRBAC. For RBAC, the TS500 and ACI 363R-10 showed moderate underestimations of 13.7% and 10.1%, respectively, while Eurocode-2 and CSA A23.3-04 presented significant underestimations of 17.9% and 42.6%, respectively. For TRBAC, TS500, ACI 363R-10, and Eurocode-2 reflected moderate underestimations of 13.1%, 12.4%, and 14.8%, respectively, while CSA A23.3-04 recorded a significant underestimation of 44.1%.

For concrete mixtures with recycled fly ash aggregates, Eurocode-2 provided a close match to the experimental means, with only slight overestimations of 2.0% for FAAC and 1.9% for TFAAC. In contrast, TS500 and ACI 363R-10 significantly overestimated the modulus of rupture by 19.9% and 22.6% for FAAC, and by 16.7% and 20.0% for TFAAC, respectively, while CSA A23.3-04 significantly underestimated the modulus of rupture by 21.7% for FAAC and 23.4% for TFAAC.

Following the detailed discussion above, Table 9 presents an overview of the codes whose prediction models show the highest correlation with the experimental results for the mechanical properties. The observed discrepancies between experimental results and code predictions stem from the fact that these standard models were empirically derived for concretes containing conventional, natural aggregates. Recycled aggregates inherently possess fundamentally different microstructures (e.g., residual adhered mortar and higher porosity) which alter crack propagation paths and generally lower the elastic modulus for a given compressive strength. Consequently, models calibrated for the stiffness of virgin rock frequently over- or underestimate the mechanical response of recycled systems. To estimate the relevant mechanical properties with greater statistical reliability, alternative linear regression models were developed based on the experimental results and are presented in Table 10. These models estimate the modulus of elasticity ($E_{c}$), splitting tensile strength ($f_{ct,sp}$), and modulus of rupture ($f_{ct,f}$) as functions of the compressive strength ($f_{cm}$) and treatment condition (T), with coefficients of determination ($R^{2}$) ranging from 0.955 to 0.969, indicating high predictive accuracy. A clear positive correlation was observed between the compressive strength and each of the other mechanical properties, confirming the consistency of the regression-based estimates with experimental trends. It must be strongly emphasized that the proposed regression models in Table 10 are empirical, in-sample fitting equations derived specifically from the limited dataset of this study, without independent cross-validation. Furthermore, due to statistical collinearity between compressive strength and the treatment variable, the multivariable regression occasionally yields negative coefficients for the treatment factor to balance the in-sample variance. Consequently, these models serve purely as descriptive mathematical summaries of the current experimental boundaries and should not be interpreted as generalized or physically mechanistic prediction tools.

## 5. Conclusions

This study presents and discusses experimental findings on the basic engineering properties of concretes produced with both treated and untreated recycled coarse aggregates derived from construction and demolition (C&D) waste streams (RCA, RBA) and an industrial by-product (FAA). To enhance the performance of recycled aggregates and by-product aggregates, a multi-step mixing technique was employed, incorporating ground granulated blast furnace slag (GGBFS) slurry, and the resulting microstructural changes were interpreted using SEM and XRD analyses. A detailed comparison was conducted between the experimental results for modulus of elasticity, splitting tensile strength, and modulus of rupture, and their corresponding values calculated using various prediction models specified in codes such as TS500, ACI, Eurocode-2, NZS 3101-1:2006, and CSA A23.3-04. This comparison aimed to assess the applicability of these models to concretes containing valorized recycled and by-product aggregates. Based on the findings, the following conclusions can be drawn.

- CSt had a lower water absorption capacity and higher specific gravity compared to recycled aggregates, which can be explained by the presence of old adhered mortar in RCA and the porous structures of RBA and FAA.
- Among the coarse aggregates, FAA had the highest ACV, followed by RBA, RCA, and CSt. Since an increase in ACV indicates that the material is mechanically more defective, CSt is the most advantageous aggregate, while FAA is the least favorable in terms of ACV.
- Fresh recycled aggregate concrete mixes were workable and cohesive without segregation. However, completely replacing the crushed stone coarse aggregate with recycled and by-product aggregates reduced the fresh density of the concrete by up to 17%, while the compressive strength values conformed to the limits for structural use, demonstrating the feasibility of C&D waste and by-product valorization in structural concrete applications.
- In terms of the mechanical properties of the concretes investigated in this study, CStC is the most favorable one, followed by TRCAC, RCAC, TRBAC, RBAC, TFAAC, and FAAC. Treating the recycled aggregates using a multi-step mixing technique led to statistically significant improvements in the mechanical properties of recycled aggregate concretes, as well as a reduction in variability, thereby enhancing statistical reliability. Although the absolute improvements are modest compared to natural aggregate concrete, the gains are highly notable from a practical standpoint, considering they were achieved at virtually zero additional operational cost through a simple mixing-based valorization process that redistributes cementitious materials, completely avoiding energy-intensive or chemically complex external pre-treatments. Moreover, considering that it utilizes standard mixing procedures, the method appears promising for potential large-scale implementation in ready-mix concrete production.
- As a result of SEM observations and quantitative XRD analyses (Rietveld refinement), it was concluded that treating the recycled and by-product aggregates increased microstructural density and strengthened the interface between aggregates and the concrete matrix. This improvement was attributed to the finer particle size of slag and enhanced pozzolanic reactions, further supporting the effectiveness of the proposed valorization approach. While the Rietveld refinement successfully provided quantitative phase analysis to support the microstructural densification claims, future studies incorporating quantitative nanoindentation to map the ITZ microhardness and advanced porosity analytics are recommended to further solidify these findings.
- The modulus of elasticity values showed the best agreement with experimental results when predicted by the CSA A23.3-04 and ACI 318-08 models for RCAC and TRCAC, respectively, with both yielding moderate underestimations. For RBAC and TRBAC, the CSA A23.3-04 model provided the closest estimates with moderate underestimations. For FAAC and TFAAC, the Eurocode-2 model demonstrated the most consistent performance, exhibiting moderate overestimations.
- The splitting tensile strength values were best captured by the TS500 and NZS 3101-1:2006 models for RBAC, with negligible and slight underestimations, respectively. For TRBAC, the TS500 model provided the closest prediction. For FAAC and TFAAC, the NZS 3101-1:2006 model yielded the most accurate results, showing slight deviations, while the TS500 model also performed well for both mixtures.
- When comparing the net modulus of rupture of the notched specimens against theoretical unnotched code predictions, the experimental net strengths of RCAC and TRCAC remarkably reached the ACI 363R-10 unnotched baseline with negligible deviations. For FAAC and TFAAC, the net strengths showed the closest agreement with the Eurocode-2 unnotched baseline. This suggests that despite the severe stress concentrations introduced by the notch, these specific treated concretes exhibited high defect-tolerance, achieving net strength levels comparable to standard unnotched estimations.
- This study demonstrates that certain code-based models can reasonably estimate the mechanical properties of concretes containing valorized recycled and by-product aggregates, particularly when virgin coarse aggregates are fully replaced and the control concrete is designed to achieve compressive strength levels in the range of 40–50 MPa. These findings support the potential extension of existing design codes to recycled aggregate concretes derived from C&D waste streams and industrial by-products.

However, despite these promising outcomes regarding mechanical strength and resource efficiency, future research incorporating a comprehensive evaluation of long-term properties (including durability, shrinkage, creep, and reinforcement bond behavior) as well as rigorous Life Cycle Assessment (LCA) and cost-benefit analysis is required to quantitatively establish the net environmental sustainability and long-term structural viability of these concrete mixtures.

Overall, this research highlights the high-value valorization potential of C&D waste streams and industrial by-products in eco-friendly concrete, contributing to waste diversion, resource efficiency, and reduced reliance on virgin aggregates. The outcomes provide both scientific insight and practical guidance for material design and potential structural applications, while also supporting the development of future design standards and facilitating the pathway toward large-scale adoption of these resource-efficient concrete technologies.

## Figures and Tables

**Table 1:** Physical characteristics and chemical constituents of the cement, slag, and fly ash (wt.%).

Oxide (%)	Cement	GGBFS	Fly Ash
SiO_2_	19.80	40.95	54.76
Al_2_O_3_	5.58	12.10	25.26
Fe_2_O_3_	3.42	1.28	6.28
CaO	63.70	36.63	2.10
MgO	1.22	5.48	2.08
SO_3_	3.34	0.16	0.20
Na_2_O	0.24	0.56	0.38
K_2_O	0.66	0.36	4.04
Cl^−^	0.04	0.018	0.0043
Loss on ignition	1.85	0.11	3.30
Insoluble residue	0.29	-	-
Free CaO	2.20	-	-
Density (g/cm^3^)	3.14	2.95	2.13
Specific surface (cm^2^/g)	3490	5253	2860
Residue on 45 µm sieve (%)	4.5	1.4	29.4
Residue on 90 µm sieve (%)	0.3	0.0	-

**Table 2:** Properties of superplasticizer.

Properties	Superplasticizer Used
Color	Dark Brown
Physical State	Liquid
Odor	Musty
Specific Gravity	1.21
pH	Approx. 7.0
Chloride Content	≤0.1% (BS EN 480-10 [118])
Alkali Content	≤5% (BS EN 480-12 [119])
Water Solubility	Completely Soluble

**Table 3:** Concrete mix proportions.

Concrete Mixtures	Cement (kg/m^3^)	GGBFS (kg/m^3^)	Water (kg/m^3^)	Fine Aggregates (kg/m^3^)		Coarse Aggregates (kg/m^3^)								SP
				River Sand	Crushed Sand	Crushed Stone		RCA		RBA		FAA		% by Binder Mass
						No. I	No. II	No. I	No. II	No. I	No. II	No. I	No. II	
CStC	321	129	212	85	693	477	477	-	-	-	-	-	-	0.85
RCAC	321	129	212	85	693	-	-	424	429	-	-	-	-	0.75
TRCAC	321	129	212	85	693	-	-	424	429	-	-	-	-	
RBAC	321	129	212	85	693	-	-	-	-	367	371	-	-	0.65
TRBAC	321	129	212	85	693	-	-	-	-	367	371	-	-	
FAAC	321	129	212	85	693	-	-	-	-	-	-	291	295	0.55
TFAAC	321	129	212	85	693	-	-	-	-	-	-	291	298	

**Table 4:** Properties of coarse aggregates.

Properties	FAA		RBA		RCA		CSt	
	No. I	No. II	No. I	No. II	No. I	No. II	No. I	No. II
Unit Weight, (kg/m^3^)	890	910	1030	1040	1370	1440	1650	1650
Specific Gravity (SSD)	1.65	1.67	2.08	2.10	2.40	2.43	2.70	2.70
Water Absorption, %	21.70	19.10	13.50	11.54	6.65	3.04	0.65	0.54
Flakiness Index (FI), %	0.1		6.1		11.7		16.4	
Aggregate Crushing Value (ACV), %	45.9		39.9		24.1		12.1	

**Table 8:** Prediction models proposed by different codes.

Mechanical Properties	Codes	Prediction Models	Units
Modulus of elasticity	TS500	$Ec=3250\sqrt{f_{ck}}+14000$	$f_{ck}:(MPa)\;Ec:(MPa)$
	ACI 318-08	$Ec=4700\sqrt{f_{c}^{\prime }}$	$f_{c}^{\prime }:(MPa)\;Ec:(MPa)$
		^a^ $Ec=4700\times 0.85\times \sqrt{f_{c}^{\prime }}$	$f_{c}^{\prime }:(MPa)\;Ec:(MPa)$
	Eurocode-2	$Ec=22{\left(f_{cm}/10\right)}^{0.3}$	$f_{cm}:(MPa)\;Ec:(GPa)$
		^b^ $Ec=22{\left(f_{cm}/10\right)}^{0.3}({2000/2200)}^{2}$	$f_{cm}:(MPa)\;Ec:(GPa)$
	CSA A23.3-04	$Ec=(3300\sqrt{f_{c}^{\prime }}+6900)({\gamma_{c}/2300)}^{1.5}$	$f_{c}^{\prime }:\left(MPa\right)\;Ec:\left(MPa\right)\;\gamma_{c}:(kg/m^{3})$
Splitting tensile strength	TS500	$f_{ct,sp}=0.525\sqrt{f_{ck}}$	$f_{ct,sp}:(MPa)\;f_{ck}:(MPa)$
	ACI 363R-10	$f_{ct,sp}=0.59\sqrt{f_{c}^{\prime }}$	$f_{ct,sp}:(MPa)\;f_{cm}:(MPa)$
		^a^ $f_{ct,sp}=0.59\times 0.85\times \sqrt{f_{c}^{\prime }}$	$f_{ct,sp}:(MPa)\;f_{cm}:(MPa)$
	Eurocode-2	$f_{ct,sp}=1/3{\left(f_{cm}-8\;\right)}^{2/3}$	$f_{ct,sp}:(MPa)\;f_{cm}:(MPa)$
		^b^ $f_{ct,sp}=1/3{\left(f_{cm}-8\;\right)}^{2/3}\left(0.4+0.6*2000/2200\right)$	$f_{ct,sp}:(MPa)\;f_{cm}:(MPa)$
	NZS 3101-1:2006	$f_{ct,sp}=0.67\sqrt{f_{c}^{\prime }}$	$f_{ct,sp}:(MPa)\;f_{c}^{\prime }:(MPa)$
		^a^ $f_{ct,sp}=0.67\times 0.85\times \sqrt{f_{c}^{\prime }}$	$f_{ct,sp}:(MPa)\;f_{c}^{\prime }:(MPa)$
Modulus of rupture	TS500	$f_{ct,f}=0.7\sqrt{f_{ck}}$	$f_{ct,f}:(MPa)\;f_{ck}:(MPa)$
	ACI 363R-10	$f_{ct,f}=0.94\sqrt{f_{c}^{\prime }}$	$f_{ct,f}:(MPa)\;f_{cm}:(MPa)$
		^a^ $f_{ct,f}=0.94\times 0.85\times \sqrt{f_{c}^{\prime }}$	$f_{ct,f}:(MPa)\;f_{cm}:(MPa)$
	Eurocode-2	$f_{ct,f}=0.459{\left(f_{cm}-8\;MPa\right)}^{2/3}$	$f_{ct,f}:(MPa)\;f_{cm}:(MPa)$
		^b^ $f_{ct,f}=0.459{\left(f_{cm}-8\;MPa\right)}^{2/3}\left(0.4+0.6*2000/2200\right)$	$f_{ct,f}:(MPa)\;f_{cm}:(MPa)$
	CSA A23.3-04	$f_{ct,f}=0.6\sqrt{f_{c}^{\prime }}$	$f_{ct,f}:(MPa)\;f_{cm}:(MPa)$
		^c^ $f_{ct,f}=0.6\times 0.85\times \sqrt{f_{c}^{\prime }}$	$f_{ct,f}:(MPa)\;f_{cm}:(MPa)$

Ec: Modulus of elasticity of concrete at 28 days. $f_{c}^{\prime },\;f_{ck}:\;$Characteristic compressive strength of concrete at 28 days. $f_{cm}:$ Mean compressive strength of concrete at 28 days. $f_{ct,sp}:$ Splitting tensile strength of concrete at 28 days. $f_{ct,f}:$ Modulus of rupture of concrete at 28 days. ^a^ Stands for the concretes containing coarse aggregates such as FAA and RBA, which are considered lightweight aggregates based on their bulk density values. ^b^ Stands for FAAC and TFAAC regarding the concrete unit weight. ^c^ Stands for the concretes having unit weight between 1850 and 2150 kg/m^3^ regarding the related standard.

**Table 9:** Prediction models with highest correlation to experimental results.

Concretes	Modulus of Elasticity	Splitting Tensile Strength	Modulus of Rupture
CStC	TS500 (2.4%, Slight U.)	Eurocode-2 (3.1%, Slight U.)	ACI 363R-10 (17.7%, Signif. U.)
	Eurocode-2 (2.5%, Slight U.)		
TRCAC	ACI 318-08 (12.8%, M.U.)	NZS 3101-1:2006 (5.3%, M.O.)	ACI 363R-10 (0.2%, N.U.)
RCAC	ACI 318-08 (14.3%, M.U.)	ACI 363R-10 (5.8%, M.U.)	ACI 363R-10 (0.01%, N.O.)
	CSA A23.3-04 (14.3%, M.U.)		
TRBAC	CSA A23.3-04 (13.0%, M.U.)	TS500 (1.4%, Slight U.)	ACI 363R-10 (12.4%, M.U.)
RBAC	CSA A23.3-04 (10.5%, M.U.)	TS500 (0.4%, N.U.)	ACI 363R-10 (10.1%, M.U.)
		NZS 3101-1:2006 (1.3%, Slight U.)	
TFAAC	Eurocode-2 (8.3%, M.O.)	NZS 3101-1:2006 (0.7%, N.O.)	Eurocode-2 (1.9%, Slight O.)
FAAC	Eurocode-2 (9.5%, M.O.)	NZS 3101-1:2006 (1.3%, Slight O.)	Eurocode-2 (2.0%, Slight O.)

U: underestimation, O: overestimation, M: moderate, N: negligible.

**Table 10:** Estimating equations derived based on test results.

Mechanical Properties	Estimating Equations	Units	Condition
Modulus of elasticity	$Ec=7614.136+593.818x(f_{cm})-704.907x(T),\;R^{2}=0.957$	$f_{cm}:(MPa)\;Ec:(MPa)$	T = 1, If concretes incorporate treated recycled aggregates T = 0, otherwise
Splitting tensile strength	$f_{ct,sp}=-1.854+0.854x\sqrt{f_{cm}}+0.016x(T),\;R^{2}=0.969$	$f_{ct,sp}:(MPa)\;f_{cm}:(MPa)$	
Modulus of rupture	$f_{ct,f}=-7.143+2.091x\sqrt{f_{cm}}-0.224x(T),\;R^{2}=0.955$	$f_{ct,f}:(MPa)\;f_{cm}:(MPa)$	

Ec: Modulus of elasticity of concrete at 28 days. $f_{cm}:$ Mean compressive strength of concrete at 28 days. $f_{ct,sp}:$ Splitting tensile strength of concrete at 28 days. $f_{ct,f}:$ Modulus of rupture of concrete at 28 days.

## Data Availability

The original contributions presented in this study are included in the article. Further inquiries can be directed to the corresponding author.

## Appendix A

**Table A1:** Compressive strength of concretes.

Properties	Statistical Parameters	CStC	TRCAC	RCAC	TRBAC	RBAC	TFAAC	FAAC
Compressive Strength, (MPa)	*n*	12	14	13	13	14	13	13
	Mean (x)	45.51	33.91	31.42	29.85	27.61	25.60	23.47
	SD (σ)	3.87	2.95	3.17	1.93	2.35	2.18	2.30
	CoV (σ/x) %	8.49	8.68	10.10	6.47	8.50	8.53	9.81

*n*: number of specimens tested; SD: standard deviation; CoV: coefficient of variation.

**Table A2:** Statistical parameters for assessing the measured and predicted results of modulus of elasticity.

Concretes	Statistical Parameters	Modulus of Elasticity, (GPa)				
		Measured	Calculated			
			TS500	ACI 318-08	Eurocode-2	CSA A23.3-04
CStC	*n*	12	12	12	12	12
	Mean (x)	35.51	34.68	28.49	34.64	27.54
	SD (σ)	0.90	0.99	1.37	0.89	0.98
	CoV (σ/x) %	2.52	2.86	4.80	2.57	3.57
	x_calculated_ − x_measured_	-	−0.84	−7.02	−0.88	−7.97
	% change	-	−2.36	−19.78	−2.47	−22.45
RCAC	*n*	13	13	13	13	13
	Mean (x)	26.20	30.97	22.45	30.99	22.47
	SD (σ)	1.16	0.99	1.56	0.94	1.09
	CoV (σ/x) %	4.42	3.18	6.95	3.03	4.83
	x_calculated_ − x_measured_	-	4.77	−3.75	4.78	−3.74
	% change	-	18.21	−14.31	18.26	−14.26
TRCAC	*n*	14	14	14	14	14
	Mean (x)	27.14	31.82	23.66	31.71	23.30
	SD (σ)	0.82	0.87	1.37	0.82	0.95
	CoV (σ/x) %	3.03	2.73	5.78	2.60	4.08
	x_calculated_ − x_measured_	-	4.69	−3.48	4.57	−3.83
	% change	-	17.27	−12.82	16.86	−14.12
RBAC	*n*	14	14	14	14	14
	Mean (x)	23.44	30.10	18.11	29.81	20.99
	SD (σ)	0.79	0.76	1.01	0.75	0.80
	CoV (σ/x) %	3.36	2.51	5.60	2.50	3.83
	x_calculated_ − x_measured_	-	6.67	−5.33	6.38	−2.45
	% change	-	28.46	−22.73	27.22	−10.45
TRBAC	*n*	13	13	13	13	13
	Mean (x)	24.41	31.00	18.44	30.53	21.25
	SD (σ)	0.67	0.59	0.82	0.58	0.65
	CoV (σ/x) %	2.75	1.91	4.47	1.92	3.08
	x_calculated_ − x_measured_	-	6.59	−5.97	6.12	−3.16
	% change	-	26.98	−24.45	25.07	−12.95
FAAC	*n*	13	13	13	13	13
	Mean (x)	21.43	28.70	16.18	23.46	18.90
	SD (σ)	0.75	0.83	1.15	0.70	0.88
	CoV (σ/x) %	3.51	2.90	7.09	2.98	4.67
	x_calculated_ − x_measured_	-	7.27	−5.25	2.03	−2.54
	% change	-	33.93	−24.51	9.48	−11.83
TFAAC	*n*	13	13	13	13	13
	Mean (x)	22.24	29.51	17.20	24.09	19.69
	SD (σ)	0.66	0.74	1.01	0.61	0.77
	CoV (σ/x) %	2.97	2.51	5.85	2.55	3.94
	x_calculated_ − x_measured_	-	7.26	−5.04	1.85	−2.56
	% change	-	32.66	−22.65	8.31	−11.49

*n*: number of specimens tested; SD: standard deviation; CoV: coefficient of variation.

**Table A3:** Statistical parameters for assessing the measured and predicted results of splitting tensile strength.

Concretes	Statistical Parameters	Splitting Tensile Strength, (MPa)				
		Measured	Calculated			
			TS500	ACI 363R-10	Eurocode-2	NZS 3101-1:2006
CStC	*n*	12	12	12	12	12
	Mean (x)	3.85	3.34	3.58	3.73	4.06
	SD (σ)	0.17	0.16	0.17	0.26	0.19
	CoV (σ/x) %	4.47	4.79	4.80	6.90	4.80
	x_calculated_ − x_measured_	-	−0.51	−0.27	−0.12	0.21
	% change	-	−13.23	−7.09	−3.08	5.51
RCAC	*n*	13	13	13	13	13
	Mean (x)	2.99	2.74	2.82	2.72	3.20
	SD (σ)	0.31	0.16	0.20	0.25	0.22
	CoV (σ/x) %	10.36	5.81	6.95	9.05	6.95
	x_calculated_ − x_measured_	-	−0.25	−0.17	−0.27	0.21
	% change	-	−8.31	−5.75	−8.92	7.03
TRCAC	*n*	13	13	13	13	13
	Mean (x)	3.18	2.86	2.95	2.89	3.35
	SD (σ)	0.23	0.13	0.16	0.20	0.18
	CoV (σ/x) %	7.07	4.50	5.35	7.00	5.35
	x_calculated_ − x_measured_	-	−0.32	−0.23	−0.29	0.17
	% change	-	−10.04	−7.31	−9.23	5.25
RBAC	*n*	13	13	13	13	13
	Mean (x)	2.59	2.58	2.25	2.39	2.56
	SD (σ)	0.21	0.11	0.11	0.17	0.13
	CoV (σ/x) %	8.24	4.13	4.93	6.96	4.93
	x_calculated_ − x_measured_	-	−0.01	−0.34	−0.20	−0.03
	% change	-	−0.41	−13.09	−7.78	−1.30
TRBAC	*n*	12	12	12	12	12
	Mean (x)	2.77	2.73	2.30	2.58	2.61
	SD (σ)	0.20	0.08	0.09	0.13	0.10
	CoV (σ/x) %	7.32	2.97	3.81	4.98	3.81
	x_calculated_ − x_measured_	-	−0.04	−0.47	−0.19	−0.16
	% change	-	−1.41	−17.00	−6.89	−5.75
FAAC	*n*	13	13	13	13	13
	Mean (x)	2.28	2.38	2.03	1.95	2.31
	SD (σ)	0.20	0.13	0.14	0.20	0.16
	CoV (σ/x) %	9.00	5.67	7.09	10.02	7.09
	x_calculated_ − x_measured_	-	0.10	−0.25	−0.32	0.03
	% change	-	4.35	−10.78	−14.22	1.32
TFAAC	*n*	13	13	13	13	13
	Mean (x)	2.44	2.50	2.16	2.13	2.45
	SD (σ)	0.18	0.12	0.13	0.18	0.14
	CoV (σ/x) %	7.41	4.77	5.85	8.25	5.85
	x_calculated_ − x_measured_	-	0.07	−0.28	−0.31	0.02
	% change	-	2.81	−11.35	−12.59	0.67

*n*: number of specimens tested; SD: standard deviation; CoV: coefficient of variation.

**Table A4:** Statistical parameters for assessing the measured and predicted results of modulus of rupture.

Concretes	Statistical Parameters	Modulus of Rupture, (MPa)				
		Measured	Calculated			
			TS500	ACI 363R-10	Eurocode-2	CSA A23.3-04
CStC	*n*	12	12	12	12	12
	Mean (x)	6.93	4.45	5.70	5.14	3.64
	SD (σ)	0.63	0.21	0.27	0.35	0.17
	CoV (σ/x) %	9.09	4.79	4.80	6.90	4.80
	x_calculated_ − x_measured_	-	−2.47	−1.23	−1.79	−3.29
	% change	-	−35.70	−17.72	−25.82	−47.48
RCAC	*n*	12	12	12	12	12
	Mean (x)	4.51	3.67	4.51	3.77	2.88
	SD (σ)	0.28	0.22	0.32	0.34	0.20
	CoV (σ/x) %	6.17	5.86	7.01	9.11	7.01
	x_calculated_ − x_measured_	-	−0.84	0.00	−0.74	−1.63
	% change	-	−18.64	0.01	−16.35	−36.16
TRCAC	*n*	12	12	12	12	12
	Mean (x)	4.74	3.83	4.72	4.01	3.02
	SD (σ)	0.27	0.16	0.24	0.27	0.15
	CoV (σ/x) %	5.76	4.30	5.10	6.68	5.10
	x_calculated_ − x_measured_	-	−0.90	−0.01	−0.73	−1.72
	% change	-	−19.04	−0.24	−15.41	−36.32
RBAC	*n*	12	12	12	12	12
	Mean (x)	3.96	3.42	3.56	3.25	2.27
	SD (σ)	0.20	0.11	0.14	0.18	0.09
	CoV (σ/x) %	5.17	3.29	3.95	5.56	3.95
	x_calculated_ − x_measured_	-	−0.54	−0.40	−0.71	−1.69
	% change	-	−13.67	−10.10	−17.87	−42.62
TRBAC	*n*	12	12	12	12	12
	Mean (x)	4.22	3.67	3.70	3.59	2.36
	SD (σ)	0.21	0.13	0.17	0.22	0.11
	CoV (σ/x) %	5.00	3.59	4.60	6.01	4.60
	x_calculated_ − x_measured_	-	−0.55	−0.52	−0.63	−1.86
	% change	-	−13.13	−12.43	−14.84	−44.10
FAAC	*n*	12	12	12	12	12
	Mean (x)	2.65	3.17	3.24	2.70	2.07
	SD (σ)	0.23	0.19	0.24	0.28	0.15
	CoV (σ/x) %	8.71	5.84	7.31	10.32	7.31
	x_calculated_ − x_measured_	-	0.53	0.60	0.05	−0.58
	% change	-	19.94	22.61	2.01	−21.74
TFAAC	*n*	12	12	12	12	12
	Mean (x)	2.84	3.32	3.41	2.90	2.18
	SD (σ)	0.20	0.14	0.18	0.21	0.11
	CoV (σ/x) %	6.86	4.25	5.23	7.39	5.23
	x_calculated_ − x_measured_	-	0.47	0.57	0.05	−0.66
	% change	-	16.69	20.04	1.92	−23.38

*n*: number of specimens tested; SD: standard deviation; CoV: coefficient of variation.

## References

[1] Akhtar A., Sarmah A.K. (2018). Construction and Demolition Waste Generation and Properties of Recycled Aggregate Concrete: A Global Perspective. J. Clean. Prod..

[2] Antunes A., Silvestre J., Costa H., do Carmo R., Júlio E. (2024). Reducing the environmental impact of the end-of-life of buildings depending on interrelated demolition strategies, transport distances and disposal scenarios. J. Build. Eng..

[3] Wang L., Zhu Z., Xie X., Wu J. (2024). Research trends in the treatment and recycling of construction and demolition waste based on literature data-driven visualization. J. Environ. Manag..

[4] Trancone G., Policastro G., Spasiano D., Race M., Parrino F., Fratino U., Fabbricino M., Pirozzi F. (2025). Treatment of concrete waste from construction and demolition activities: Application of organic acids from continuous dark fermentation in moving bed biofilm reactors. Chem. Eng. J..

[5] Khandani F.S., Atapour H., Yousefi Rad M., Khosh B. (2023). An experimental study on the mechanical properties of under-ground mining backfill materials obtained from recycling of construction and demolition waste. Case Stud. Constr. Mater..

[6] Zhang L.W., Sojobi A.O., Kodur V.K.R., Liew K.M. (2019). Effective Utilization and Recycling of Mixed Recycled Aggregates for a Greener Environment. J. Clean. Prod..

[7] Cardoza A., Colorado H.A. (2023). Alkali-activated cement manufactured by the alkaline activation of demolition and construction waste using brick and concrete wastes. Open Ceram..

[8] Gomes N.T., Tavares K.M.S., Barroso L.S., Xavier G.C., de Azevedo A.R.G., Monteiro S.N., Ribeiro R.P. (2026). Application of construction and demolition waste in mortars and after heat treatment: A review. J. Mater. Res. Technol..

[9] Tanthanawiwat K., Gheewala S.H., Nilsalab P., Schoch M., Silalertruksa T. (2024). Environmental sustainability and cost performances of construction and demolition waste management scenarios: A case study of timber and concrete houses in Thailand. J. Clean. Prod..

[10] Ozcelikci E., Hu M., van der Meide M., Sahmaran M. (2025). Life cycle assessment of SCM substitution in various CDW-based geopolymer concretes and sensitivity analyses on allocation methods. Waste Manag..

[11] Safi O., Ibrahim H., Cousture A., Ghorbel E., Wardeh G. (2026). The impact of combined recycled coarse aggregates from non-hazardous construction and demolition waste on self-compacting concrete performance for sustainable construction. Clean. Waste Syst..

[12] Ozturk O., Yildirim H., Ozyurt N., Ozturan T. (2022). Evaluation of Mechanical Properties and Structural Behaviour of Concrete Pavements Produced with Virgin and Recycled Aggregates: An Experimental and Numerical Study. Int. J. Pavement Eng..

[13] Shi X., Mirsayar M.M., Mukhopadhyay A., Zollinger D. (2019). Characterization of Two-Parameter Fracture Properties of Portland Cement Concrete Containing Reclaimed Asphalt Pavement Aggregates by Semicircular Bending Specimens. Cem. Concr. Compos..

[14] Zheng C., Lou C., Du G., Li X., Liu Z., Li L. (2018). Mechanical Properties of Recycled Concrete with Demolished Waste Concrete Aggregate and Clay Brick Aggregate. Results Phys..

[15] Munir M.J., Kazmi S.M.S., Wu Y.F., Hanif A., Khan M.U.A. (2018). Thermally Efficient Fired Clay Bricks Incorporating Waste Marble Sludge: An Industrial-Scale Study. J. Clean. Prod..

[16] Wang B., Yan L., Fu Q., Kasal B. (2021). A Comprehensive Review on Recycled Aggregate and Recycled Aggregate Concrete. Resour. Conserv. Recycl..

[17] Yildirim H., Özturan T. Modulus of Rupture of Recycled Aggregate Concrete. Proceedings of the International Conference on Agriculture, Technology, Engineering and Sciences (ICATES 2018).

[18] Ozturk O., Yildirim H., Ozyurt N., Ozturan T. (2022). Mechanical Properties and Structural Requirements of Recycled Aggregate Concrete for Pavements. Eng. Proc..

[19] Ozturk O., Yildirim H., Ozyurt N., Ozturan T. (2025). Post-Cracking Cyclic Shear Performance of Recycled Aggregate Concrete for Pavements. Transp. Res. Rec..

[20] Letelier V., Hott F., Bustamante M., Wenzel B. (2024). Effect of recycled coarse aggregate treated with recycled binder paste coating and accelerated carbonation on mechanical and physical properties of concrete. J. Build. Eng..

[21] Gangu S.K., Shankar S. (2024). Recycled Concrete Aggregate Stabilized with Lime-Fly Ash and Cement for Utilization as a Semirigid Base Course of Low-Volume Roads. J. Mater. Civ. Eng..

[22] Atasham ul haq M., Wang P., Gong F., Hassam M., Iqbal M.S., Li W., Tahir M. (2026). Influence of agricultural waste ash slurry-treated recycled brick aggregates on the mechanical and durability performance of concrete. Case Stud. Constr. Mater..

[23] Brasileiro K.P.T.V., de Oliveira Nahime B., Lima E.C., Alves M.M., Ferreira W.P., dos Santos I.S., Bezerra Filho C.P., dos Reis I.C. (2024). Influence of recycled aggregates and silica fume on the performance of pervious concrete. J. Build. Eng..

[24] Munir Q., Lahtela V., Rasilainen I., Kärki T., Khan M.M.H. (2026). Optimizing landfilled construction and demolition waste fractions for concrete: Recycling, design, and cost analysis. Sustain. Horiz..

[25] Mahmoodi O., Siad H., Lachemi M., Şahmaran M. (2026). Structural performance of reinforced geopolymer concrete beams incorporating construction and demolition waste-based recycled precursors and aggregates. Structures.

[26] Zhang S., He P., Niu L. (2020). Mechanical Properties and Permeability of Fiber-Reinforced Concrete with Recycled Aggregate Made from Waste Clay Brick. J. Clean. Prod..

[27] Rashid K., Rehman M.U., de Brito J., Ghafoor H. (2020). Multi-Criteria Optimization of Recycled Aggregate Concrete Mixes. J. Clean. Prod..

[28] Tam V.W.Y., Tam C.M., Le K.N. (2007). Removal of Cement Mortar Remains from Recycled Aggregate Using Pre-Soaking Approaches. Resour. Conserv. Recycl..

[29] Kim Y., Hanif A., Kazmi S.M.S., Munir M.J., Park C. (2018). Properties Enhancement of Recycled Aggregate Concrete Through Pretreatment of Coarse Aggregates—Comparative Assessment of Assorted Techniques. J. Clean. Prod..

[30] Pawluczuk E., Kalinowska-Wichrowska K., Bołtryk M., Jiménez J.R., Fernández J.M. (2019). The Influence of Heat and Mechanical Treatment of Concrete Rubble on the Properties of Recycled Aggregate Concrete. Materials.

[31] Purushothaman R., Amirthavalli R.R., Karan L. (2014). Influence of Treatment Methods on the Strength and Performance Characteristics of Recycled Aggregate Concrete. J. Mater. Civ. Eng..

[32] Babu V.S., Mullick A.K., Jain K.K., Singh P.K. (2014). Strength and Durability Characteristics of High-Strength Concrete with Recycled Aggregate-Influence of Processing. J. Sustain. Cem. Based Mater..

[33] Katz A. (2004). Treatments for the Improvement of Recycled Aggregate. J. Mater. Civ. Eng..

[34] Dilbas H., Çakır Ö., Yıldırım H. (2020). An Experimental Investigation on Fracture Parameters of Recycled Aggregate Concrete with Optimized Ball Milling Method. Constr. Build. Mater..

[35] Kazemian F., Rooholamini H., Hassani A. (2019). Mechanical and Fracture Properties of Concrete Containing Treated and Untreated Recycled Concrete Aggregates. Constr. Build. Mater..

[36] Güneyisi E., Gesoǧlu M., Algin Z., Yazici H. (2014). Effect of Surface Treatment Methods on the Properties of Self-Compacting Concrete with Recycled Aggregates. Constr. Build. Mater..

[37] Verma A., Babu V.S., Arunachalam S. (2022). Influence of Modified Two-Stage Mixing Approaches on Recycled Aggregate Treated with a Hybrid Method of Treatment. Aust. J. Struct. Eng..

[38] Tsujino M., Noguchi T., Tamura M., Kanematsu M., Maruyama I. (2007). Application of Conventionally Recycled Coarse Aggregate to Concrete Structure by Surface Modification Treatment. J. Adv. Concr. Technol..

[39] Kou S.C., Poon C.S. (2010). Properties of Concrete Prepared with PVA-Impregnated Recycled Concrete Aggregates. Cem. Concr. Compos..

[40] Shi C., Wu Z., Cao Z., Ling T.C., Zheng J. (2018). Performance of Mortar Prepared with Recycled Concrete Aggregate Enhanced by CO_2_ and Pozzolan Slurry. Cem. Concr. Compos..

[41] Spaeth V., Tegguer A.D. (2013). Improvement of Recycled Concrete Aggregate Properties by Polymer Treatments. Int. J. Sustain. Built Environ..

[42] Shaban W.M., Elbaz K., Yang J., Thomas B.S., Shen X., Li L., Du Y., Xie J., Li L. (2021). Effect of Pozzolan Slurries on Recycled Aggregate Concrete: Mechanical and Durability Performance. Constr. Build. Mater..

[43] Velardo P., Sáez del Bosque I.F., Matías A., Sánchez de Rojas M.I., Medina C. (2021). Properties of Concretes Bearing Mixed Recycled Aggregate with Polymer-Modified Surfaces. J. Build. Eng..

[44] Liang Y., Ye Z., Vernerey F., Xi Y. (2015). Development of Processing Methods to Improve Strength of Concrete with 100% Recycled Coarse Aggregate. J. Mater. Civ. Eng..

[45] Wang J., Zhang J., Cao D., Dang H., Ding B. (2020). Comparison of Recycled Aggregate Treatment Methods on the Performance for Recycled Concrete. Constr. Build. Mater..

[46] Sasanipour H., Aslani F., Taherinezhad J. (2021). Chloride Ion Permeability Improvement of Recycled Aggregate Concrete Using Pretreated Recycled Aggregates by Silica Fume Slurry. Constr. Build. Mater..

[47] Kisku N., Rajhans P., Panda S.K., Pandey V., Nayak S. (2020). Microstructural Investigation of Recycled Aggregate Concrete Produced by Adopting Equal Mortar Volume Method along with Two Stage Mixing Approach. Structures.

[48] Gao C., Huang L., Yan L., Jin R., Chen H. (2020). Mechanical Properties of Recycled Aggregate Concrete Modified by Nano-Particles. Constr. Build. Mater..

[49] Bui N.K., Satomi T., Takahashi H. (2019). Influence of Industrial By-Products and Waste Paper Sludge Ash on Properties of Recycled Aggregate Concrete. J. Clean. Prod..

[50] Yue Y., Zhou Y., Xing F., Gong G., Hu B., Guo M. (2020). An Industrial Applicable Method to Improve the Properties of Recycled Aggregate Concrete by Incorporating Nano-Silica and Micro-CaCO_3_. J. Clean. Prod..

[51] Tam V.W.Y., Tam C.M. (2008). Diversifying Two-Stage Mixing Approach (TSMA) for Recycled Aggregate Concrete: TSMAs and TSMAsc. Constr. Build. Mater..

[52] Tam V.W.Y., Tam C.M. (2007). Assessment of Durability of Recycled Aggregate Concrete Produced by Two-Stage Mixing Approach. J. Mater. Sci..

[53] Tam V.W.Y., Gao X.F., Tam C.M. (2005). Microstructural Analysis of Recycled Aggregate Concrete Produced from Two-Stage Mixing Approach. Cem. Concr. Res..

[54] Babu V.S., Mullick A.K., Jain K.K., Singh P.K. (2014). Strength and Durability Characteristics of High-Strength Concrete with Recycled Aggregate—Influence of Mixing Techniques. J. Sustain. Cem. Based Mater..

[55] Kisku N., Rajhans P., Panda S.K., Nayak S., Pandey V. (2020). Development of Durable Concrete from C&D Waste by Adopting Identical Mortar Volume Method in Conjunction with Two-Stage Mixing Procedure. Constr. Build. Mater..

[56] Pradhan S., Kumar S., Barai S.V. (2017). Recycled Aggregate Concrete: Particle Packing Method (PPM) of Mix Design Approach. Constr. Build. Mater..

[57] Kong D., Lei T., Zheng J., Ma C., Jiang J., Jiang J. (2010). Effect and Mechanism of Surface-Coating Pozzalanics Materials Around Aggregate on Properties and ITZ Microstructure of Recycled Aggregate Concrete. Constr. Build. Mater..

[58] Li J., Xiao H., Zhou Y. (2009). Influence of Coating Recycled Aggregate Surface with Pozzolanic Powder on Properties of Recycled Aggregate Concrete. Constr. Build. Mater..

[59] Ding Y., Wu J., Xu P., Zhang X., Fan Y. (2021). Treatment Methods for the Quality Improvement of Recycled Concrete Aggregate (RCA)—A Review. J. Wuhan Univ. Technol. Mater. Sci. Ed..

[60] Tam V.W.Y., Soomro M., Evangelista A.C.J. (2021). Quality Improvement of Recycled Concrete Aggregate by Removal of Residual Mortar: A Comprehensive Review of Approaches Adopted. Constr. Build. Mater..

[61] Shaban W.M., Yang J., Su H., Mo K.H., Li L., Xie J. (2019). Quality Improvement Techniques for Recycled Concrete Aggregate: A Review. J. Adv. Concr. Technol..

[62] Makul N. (2021). A Review on Methods to Improve the Quality of Recycled Concrete Aggregates. J. Sustain. Cem. Based Mater..

[63] Tang P., Florea M.V.A., Brouwers H.J.H. (2017). Employing Cold Bonded Pelletization to Produce Lightweight Aggregates from Incineration Fine Bottom Ash. J. Clean. Prod..

[64] Bhattacharjee U., Kandpal T.C. (2002). Potential of Fly Ash Utilisation in India. Energy.

[65] Das B., Prakash S., Reddy P.S.R., Misra V.N. (2007). An Overview of Utilization of Slag and Sludge from Steel Industries. Resour. Conserv. Recycl..

[66] Zhang X., Chen J., Jiang J.J., Li J., Tyagi R.D., Surampalli R.Y. (2020). The Potential Utilization of Slag Generated from Iron- and Steelmaking Industries: A Review. Environ. Geochem. Health.

[67] Xu G., Shi X. (2018). Characteristics and Applications of Fly Ash as a Sustainable Construction Material: A State-of-the-Art Review. Resour. Conserv. Recycl..

[68] Wang X., Li X., Yan X., Tu C., Yu Z. (2021). Environmental Risks for Application of Iron and Steel Slags in Soils in China: A Review. Pedosphere.

[69] Chen J., Yan B., Li H., Li P., Guo H. (2018). Vitrification of Blast Furnace Slag and Fluorite Tailings for Giving Diopside-Fluorapatite Glass-Ceramics. Mater. Lett..

[70] Ahmad J., Kontoleon K.J., Majdi A., Naqash M.T., Deifalla A.F., Kahla N.B., Isleem H.F., Qaidi S.M.A. (2022). A Comprehensive Review on the Ground Granulated Blast Furnace Slag (GGBS) in Concrete Production. Sustainability.

[71] Buddhdev B.G., Timani K.L., Gali M.L., Rao P.R. (2021). Critical Review for Utilization of Blast Furnace Slag in Geotechnical Application. Problematic Soils and Geoenvironmental Concerns.

[72] Giergiczny Z. (2019). Fly Ash and Slag. Cem. Concr. Res..

[73] Li L., Ling T.C., Pan S.Y. (2022). Environmental Benefit Assessment of Steel Slag Utilization and Carbonation: A Systematic Review. Sci. Total Environ..

[74] Huang D., Chen P., Peng H., Yuan Q., Tian X. (2022). Drying Shrinkage Performance of Medium-Ca Alkali-Activated Fly Ash and Slag Pastes. Cem. Concr. Compos..

[75] Zakira U., Zheng K., Xie N., Birgisson B. (2023). Development of High-Strength Geopolymers from Red Mud and Blast Furnace Slag. J. Clean. Prod..

[76] Jeong Y., Yum W.S., Jeon D., Oh J.E. (2017). Strength Development and Microstructural Characteristics of Barium Hydroxide-Activated Ground Granulated Blast Furnace Slag. Cem. Concr. Compos..

[77] Oge M., Ozkan D., Celik M.B., Gok M.S., Karaoglanli A.C. (2019). An Overview of Utilization of Blast Furnace and Steelmaking Slag in Various Applications. Mater. Today Proc..

[78] Baykal G., Döven A.G. (2000). Utilization of Fly Ash by Pelletization Process; Theory, Application Areas and Research Results. Resour. Conserv. Recycl..

[79] Siddique R., Cachim P. (2018). Waste and Supplementary Cementitious Materials in Concrete: Characterisation, Properties and Applications.

[80] Matthes W., Vollpracht A., Villagrán Y., Kamali-Bernard S., Hooton D., Gruyaert E., Soutsos M., Belie N.D., Belie N.D., Soutsos M., Gruyaert E. (2018). Ground Granulated Blast-Furnace Slag. Properties of Fresh and Hardened Concrete Containing Supplementary Cementitious Materials.

[81] Sideris K., Justnes H., Soutsos M., Sui T., Belie N.D., Soutsos M., Gruyaert E. (2018). Fly Ash. Properties of Fresh and Hardened Concrete Containing Supplementary Cementitious Materials.

[82] Sobolev K., Kozhukhova M., Sideris K., Menéndez E., Santhanam M., Belie N.D., Soutsos M., Gruyaert E. (2018). Alternative Supplementary Cementitious Materials. Properties of Fresh and Hardened Concrete Containing Supplementary Cementitious Materials.

[83] Zuo Y., Nedeljković M., Ye G. (2019). Pore Solution Composition of Alkali-Activated Slag/Fly Ash Pastes. Cem. Concr. Res..

[84] Das S.K., Tripathi A.K., Kandi S.K., Mustakim S.M., Bhoi B., Rajput P. (2023). Ferrochrome Slag: A Critical Review of Its Properties, Environmental Issues and Sustainable Utilization. J. Environ. Manag..

[85] Verma C.L., Handa S.K., Jain S.K., Yadav R.K. (1998). Techno-Commercial Perspective Study for Sintered Fly Ash Light-Weight Aggregates in India. Constr. Build. Mater..

[86] Kayali O., Haque M.N., Zhu B. (2003). Some Characteristics of High Strength Fiber Reinforced Lightweight Aggregate Concrete. Cem. Concr. Compos..

[87] Zhang M.H., Gjorv O.E. (1991). Characteristics of Lightweight Aggregates for High-Strength Concrete. ACI Mater. J..

[88] Kayali O. (2008). Fly Ash Lightweight Aggregates in High Performance Concrete. Constr. Build. Mater..

[89] Kockal N.U., Ozturan T. (2010). Effects of Lightweight Fly Ash Aggregate Properties on the Behavior of Lightweight Concretes. J. Hazard. Mater..

[90] Rasheed R., Javed H., Rizwan A., Sharif F., Yasar A., Tabinda A.B., Ahmad S.R., Wang Y., Su Y. (2021). Life Cycle Assessment of A Cleaner Supercritical Coal-Fired Power Plant. J. Clean. Prod..

[91] Babbitt C.W., Lindner A.S. (2005). A Life Cycle Inventory of Coal Used for Electricity Production in Florida. J. Clean. Prod..

[92] Manikandan R., Ramamurthy K. (2007). Influence of Fineness of Fly Ash on the Aggregate Pelletization Process. Cem. Concr. Compos..

[93] Harikrishnan K.I., Ramamurthy K. (2006). Influence of Pelletization Process on the Properties of Fly Ash Aggregates. Waste Manag..

[94] Gesoǧlu M., Özturan T., Güneyisi E. (2007). Effects of Fly Ash Properties on Characteristics of Cold-Bonded Fly Ash Light-weight Aggregates. Constr. Build. Mater..

[95] Rivera F., Martínez P., Castro J., López M. (2015). Massive Volume Fly-Ash Concrete: A More Sustainable Material with Fly Ash Replacing Cement and Aggregates. Cem. Concr. Compos..

[96] Kockal N.U., Ozturan T. (2016). Microstructural and Mineralogical Characterization of Artificially Produced Pellets for Civil Engineering Applications. J. Mater. Civ. Eng..

[97] Nadesan M.S., Dinakar P. (2017). Mix Design and Properties of Fly Ash Waste Lightweight Aggregates in Structural Light-weight Concrete. Case Stud. Constr. Mater..

[98] Narattha C., Chaipanich A. (2018). Phase Characterizations, Physical Properties and Strength of Environment-Friendly Cold-Bonded Fly Ash Lightweight Aggregates. J. Clean. Prod..

[99] Colangelo F., Messina F., Cioffi R. (2015). Recycling of MSWI Fly Ash by Means of Cementitious Double Step Cold Bonding Pelletization: Technological Assessment for the Production of Lightweight Artificial Aggregates. J. Hazard. Mater..

[100] Kockal N.U., Ozturan T. (2011). Characteristics of Lightweight Fly Ash Aggregates Produced with Different Binders and Heat Treatments. Cem. Concr. Compos..

[101] Yıldırım H., Ozturan T. Mechanical Properties of Lightweight Concrete Made with Cold Bonded Fly Ash Pellets. Proceedings of the 2nd International Balkans Conference on Challenges of Civil Engineering, BCCCE.

[102] Kockal N.U., Ozturan T. (2011). Durability of Lightweight Concretes with Lightweight Fly Ash Aggregates. Constr. Build. Mater..

[103] Colangelo F., Messina F., Palma L.D., Cioffi R. (2017). Recycling of Non-Metallic Automotive Shredder Residues and Coal Fly-Ash in Cold-Bonded Aggregates for Sustainable Concrete. Compos. Part B Eng..

[104] Gomathi P., Sivakumar A. (2015). Accelerated Curing Effects on the Mechanical Performance of Cold Bonded and Sintered Fly Ash Aggregate Concrete. Constr. Build. Mater..

[105] Gesoǧlu M., Özturan T., Güneyisi E. (2006). Effects of Cold-Bonded Fly Ash Aggregate Properties on the Shrinkage Cracking of Lightweight Concretes. Cem. Concr. Compos..

[106] Gesoğlu M., Özturan T., Güneyisi E. (2004). Shrinkage Cracking of Lightweight Concrete Made with Cold-Bonded Fly Ash Aggregates. Cem. Concr. Res..

[107] Kockal N.U., Ozturan T. (2011). Strength and Elastic Properties of Structural Lightweight Concretes. Mater. Des..

[108] Güneyisi E., Gesoglu M., Azez O.A., Öz H.Ö. (2016). Effect of Nano Silica on the Workability of Self-Compacting Concretes Having Untreated and Surface Treated Lightweight Aggregates. Constr. Build. Mater..

[109] Yıldırım H., Özturan T. (2021). Impact Resistance of Concrete Produced with Plain and Reinforced Cold-Bonded Fly Ash Aggregates. J. Build. Eng..

[110] (2000). Requirements for Design and Construction of Reinforced Concrete Structures.

[111] (2008). Building Code Requirements for Structural Concrete (ACI 318M-08) and Commentary.

[112] (2010). Report on High-Strength Concrete (ACI 363R-10).

[113] (2004). Eurocode 2: Design of Concrete Structures—Part 1-1: General Rules and Rules for Buildings.

[114] (2004). Design of Concrete Structures.

[115] (2006). Concrete Structures Standard-Part 1: The Design of Concrete Structures.

[116] (2019). Standard Specification for Coal Fly Ash and Raw or Calcined Natural Pozzolan for Use in Concrete.

[117] (2019). Standard Specification for Chemical Admixtures for Concrete.

[118] (2009). Admixtures for Concrete, Mortar and Grout—Test Methods—Part 10: Determination of Water Soluble Chloride Content.

[119] (2005). Admixtures for Concrete, Mortar and Grout—Test Methods—Part 12: Determination of the Alkali Content of Admixtures.

[120] (2017). Standard Test Method for Bulk Density (“Unit Weight”) and Voids in Aggregate.

[121] (2015). Standard Test Method for Relative Density (Specific Gravity) and Absorption of Coarse.

[122] (2012). Tests for Geometrical Properties of Aggregates-Part 3: Determination of Particle Shape—Flakiness Index.

[123] (1992). Specification for Aggregates from Natural Sources for Concrete.

[124] (1990). Testing Aggregates—Part 110: Methods of Determination of Aggregate Crushing Value (ACV).

[125] (2009). Design of Concrete Mixes.

[126] Lee H.S., Wang X.Y., Zhang L.N., Koh K.T. (2015). Analysis of the Optimum Usage of Slag for the Compressive Strength of Concrete. Materials.

[127] (2012). Complementary Turkish Standard to TS EN 206-1.

[128] (2000). Concrete-Part 1: Specification, Performance, Production and Conformity.

[129] (2019). Standard Practice for Making and Curing Concrete Test Specimens in the Laboratory.

[130] Shayan A., Xu A.M. (2003). Performance and Properties of Structural Concrete Made with Recycled Concrete Aggregate. ACI Mater. J..

[131] (2020). Standard Test Method for Slump of Hydraulic-Cement Concrete.

[132] (2017). Standard Test Method for Density (Unit Weight), Yield, and Air Content (Gravimetric) of Concrete.

[133] (2020). Standard Test Method for Compressive Strength of Cylindrical Concrete Specimens.

[134] (2017). Standard Test Method for Splitting Tensile Strength of Cylindrical Concrete Specimens.

[135] (2014). Standard Test Method for Static Modulus of Elasticity and Poisson’s Ratio of Concrete in Compression.

[136] (2003). Method of Test for Fracture Energy of Concrete by use of Notched Beam.

[137] (2015). Standard Test Method for Flexural Properties of Polymer Matrix Composite Materials.

[138] Lutterotti L. (2010). Total Pattern Fitting for the Combined Size-Strain-Stress-Texture Determination in Thin Film Diffraction. Nucl. Instrum. Methods Phys. Res. Sect. B Beam Interact. Mater. At..

[139] Butler L., West J.S., Tighe S.L. (2013). Effect of Recycled Concrete Coarse Aggregate from Multiple Sources on the Hardened Properties of Concrete with Equivalent Compressive Strength. Constr. Build. Mater..

[140] Yang J., Du Q., Bao Y. (2011). Concrete with Recycled Concrete Aggregate and Crushed Clay Bricks. Constr. Build. Mater..

[141] Gesoglu M., Güneyisi E., Öz H.Ö., Taha I., Yasemin M.T. (2015). Failure Characteristics of Self-Compacting Concretes Made with Recycled Aggregates. Constr. Build. Mater..

[142] Güneyisi E., Gesoglu M., Özturan T., Ipek S. (2015). Fracture Behavior and Mechanical Properties of Concrete with Artificial Lightweight Aggregate and Steel Fiber. Constr. Build. Mater..

[143] Güneyisi E., Gesoglu M., Ghanim H., Ipek S., Taha I. (2016). Influence of the Artificial Lightweight Aggregate on Fresh Properties and Compressive Strength of the Self-Compacting Mortars. Constr. Build. Mater..

[144] Abdulla N.A. (2015). Effect of Recycled Coarse Aggregate Type on Concrete. J. Mater. Civ. Eng..

[145] Rao M.C., Bhattacharyya S.K., Barai S.V. (2011). Behaviour of Recycled Aggregate Concrete Under Drop Weight Impact Load. Constr. Build. Mater..

[146] Prasad C.V.S.R. (2017). Light Weight Concrete Using FlyAsh Aggregate. Int. J. Innov. Technol..

[147] Ravichandra R., Dattatreya J.K., Maheshwarappa S.M. (2015). An Experimental Study on Properties of Fly ash Aggregate Comparing with Natural Aggregate. J. Civ. Eng. Environ. Technol..

[148] Eguchi K., Teranishi K., Nakagome A., Kishimoto H., Shinozaki K., Narikawa M. (2007). Application of Recycled Coarse Aggregate by Mixture to Concrete Construction. Constr. Build. Mater..

[149] Walpole R.E., Myers R.H., Myers S.L., Ye K. (2017). Probability & Statistics for Engineers & Scientists.

[150] Moore D.S., McCabe G.P., Craig B.A. (2014). Introduction to the Practice of Statistics.

[151] (2002). Building Code Requirements for Structural Concrete (ACI 318-02) and Commentary (ACI 318R-02).

[152] (2003). Guide for Structural Lightweight-Aggregate Concrete (ACI 213R-03).

[153] Tam V.W.Y., Tam C.M., Wang Y. (2007). Optimization on Proportion for Recycled Aggregate in Concrete Using Two-Stage Mixing Approach. Constr. Build. Mater..

[154] Lee S.F., Jacobsen S. (2011). Study of Interfacial Microstructure, Fracture Energy, Compressive Energy and Debonding Load of Steel Fiber-Reinforced Mortar. Mater. Struct..

[155] Erdem S., Dawson A.R., Thom N.H. (2012). Impact Load-Induced Micro-Structural Damage and Micro-Structure Associated Mechanical Response of Concrete Made with Different Surface Roughness and Porosity Aggregates. Cem. Concr. Res..

[156] Akca A.H., Zihnioǧlu N.Ö. (2013). High Performance Concrete Under Elevated Temperatures. Constr. Build. Mater..

[157] Gartner E.M., Kurtis K.E., Monteiro P.J.M. (2000). Proposed Mechanism of C-S-H Growth Tested by Soft X-Ray Microscopy. Cem. Concr. Res..

[158] Taylor H.F.W. (1992). Cement Chemistry.

[159] Neville A.M. (2011). Properties of Concrete.

